# Transcription regulator TrtA modulates oxygen stress response in *Treponema denticola*

**DOI:** 10.1128/aem.00750-25

**Published:** 2025-11-12

**Authors:** Yurie Kitamura, Yuichiro Kikuchi, Eitoyo Kokubu, Keiko Yamashita, Hideo Yonezawa, Atsushi Saito, Kazuyuki Ishihara

**Affiliations:** 1 Department of Periodontology, Tokyo Dental College 13093, Tokyo, Japan; 2 Department of Microbiology, Tokyo Dental College 13093, Tokyo, Japan; 3 Oral Health Science Center, Tokyo Dental College 13093, Tokyo, Japan; University of Nebraska-Lincoln, Lincoln, Nebraska, USA

**Keywords:** *Treponema denticola*, transcriptional regulator, dentilisin, oxygen stress, periodontitis, virulence

## Abstract

Periodontitis is caused by dysbiosis of the subgingival microbiome. *Treponema denticola* is an obligate anaerobe frequently detected in advanced periodontal lesions. However, the mechanism by which *T. denticola* evades oxygen stress remains unclear. TDE_0127 (*trtA*) encodes a potential transcriptional regulator in *T. denticola* and is upregulated in response to oxygen exposure. However, its exact function is unclear. This study aimed to investigate the involvement of TrtA in the oxygen stress response of *T. denticola. trtA* expression increased under oxygen exposure. RNA-seq analysis revealed that the expression levels of 38 genes, including that encoding internalin-related protein, were significantly upregulated under anaerobic conditions, and 153 genes, including those encoding peroxiredoxin and dentilisin, were significantly downregulated (|log_2_(mutant/wild type)| >1.5, false discovery rate <0.05) in the *trtA*-deletion mutant. Under aerobic conditions, peroxiredoxin expression was lower and the viable cell proportion was significantly lower in the *trtA-*deletion mutant than in the wild-type strain (*P* < 0.001), suggesting that TrtA is involved in oxygen resistance via peroxiredoxin regulation. In contrast, internalin-related protein expression in the *trtA-*deletion mutant increased. Inactivation of internalin-related protein reduced the intracellular invasion of gingival keratinocytes, and quantitative reverse transcription PCR analysis showed that internalin-related protein expression in the wild-type strain decreased under oxygen exposure, suggesting that it is under TrtA regulation. Although dentilisin expression was downregulated in the *trtA*-deletion mutant, direct interaction was not elucidated. These results suggest that TrtA is involved in oxygen stress resistance and intracellular invasion in *T. denticola*.

**IMPORTANCE:**

*Treponema denticola* is prevalent in advanced periodontal lesions and is involved in the pathogenesis and progression of periodontitis. During dysbiosis of the subgingival microbiome, *T. denticola* resists the effects of environmental oxygen and H_2_O_2_ released from commensal bacteria. In other periodontopathic bacteria, genes encoding master regulators of oxygen stress, such as OxyR, have been detected in the genome. However, an ortholog of such a regulator is yet to be detected in the genome of *T. denticola*. Herein, we show, for the first time, that TrtA is involved in the oxygen stress response of *T. denticola*. The expression of 153 genes was attenuated in the *trtA-*deletion mutant, indicating that TrtA influences a broad range of cellular processes. TrtA regulates the oxygen stress response in part through peroxiredoxin. In addition, internalin-related protein, which may participate in the host cell invasion by *T. denticola*, is under the control of TrtA.

## INTRODUCTION

Periodontal disease is caused by persistent infection with bacteria in dental plaque biofilm and an imbalanced host immune response against them (1, 2). The bacteria in dental plaque in advanced periodontal lesions are predominantly gram-negative anaerobes, including spirochetes (3). Dysbiosis, characterized by a shift in the subgingival plaque microbiota to one comprising multiple pathogenic bacteria, is a major etiological factor of the disease (4, 5). *Treponema denticola* is more prevalent in inflamed periodontal milieu than in healthy areas (6–8) and is a key microorganism for calculating the dysbiosis index based on machine learning analysis (9). *T. denticola* possesses several virulence factors, including dentilisin (a protease), major surface protein, and factor H binding protein B (10). Dentilisin is located on the surface of *T. denticola* and is involved in the hydrolysis of host proteins, including cytokines, the activation of complement, invasion into epithelial cells, and binding to fibrinogen (11–14). These processes are likely involved in the pathogenesis of periodontitis during dysbiosis.

Oxygen stress constitutes a major barrier to colonization by *T. denticola* in dental plaque biofilm. *T. denticola,* being an anaerobe, can be severely damaged by exposure to oxygen. OxyR and SoxR, which are master regulator genes that counteract this stress (15), have been identified in many bacteria. OxyR controls gene expression in response to oxygen stress in subgingival bacteria, including *Porphyromonas gingivalis*, *Capnocytophaga ochracea, Tannerella forsythia,* and *Prevotella intermedia* (16–19). However, orthologs of *oxyR* or *soxR* have not been detected in the genome of *T. denticola* (20). *T. denticola* employs several systems for gene regulation, such as the two-component regulatory system, cyclic di-GMP, and DNA-binding proteins, in response to environmental stress (21–23); however, the molecules responsible for oxygen stress response have not been fully clarified.

The expression of TDE_0127 (*trtA*), a potential transcriptional regulator in *T. denticola,* increased under exposure to oxygen (24). In our previous study, the expression of the TetR-type regulator OxtR1 increased in response to oxygen exposure, and the expression of *trtA* was 2.7-fold higher in the OxtR1-deficient mutant compared to that in the wild type (23). These results strongly suggest that *trtA* is also involved in the oxygen stress response. In addition, the expression of *trtA* increased in a dentilisin-deficient mutant (25), suggesting that it is associated with dentilisin expression. In the present study, we aimed to clarify the role of TrtA using a *trtA*-deficient mutant, focusing on the oxygen stress response and dentilisin expression.

## RESULTS

### Characterization of TrtA

**Fig 1:**
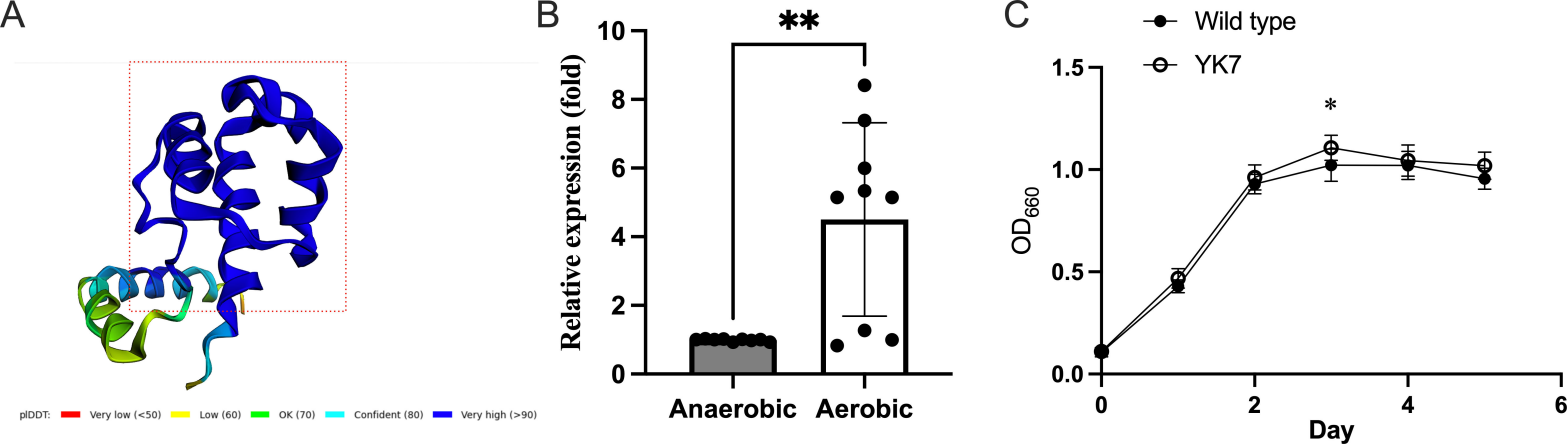
Expression of *trtA* and the growth curves for wild-type and YK7 strains. (**A**) Predicted structures of TDE_0127 via AlphaFold2. pLDDT: Predicted local distance difference test matching score. A dotted rectangle indicates the region showing similarity with the xenobiotic response element family helix-turn-helix DNA-binding domain-containing transcriptional regulator. (**B**) *T. denticola* wild-type strain was incubated under aerobic and anaerobic conditions for 1 h, and the expression of *trtA* was evaluated using real-time quantitative reverse transcription PCR (*n* = 9). (**C**) Growth curves of *T. denticola* wild-type and *trtA*-deletion mutant (YK7) strains. Wild-type (closed black circle) and YK7 (closed white circles) strains were grown at 37°C in TYGVS medium under anaerobic conditions. Data are presented as mean ± standard deviation (*n* = 15), and statistically significant differences are indicated using asterisks (unpaired t-test, *P < 0.05, **P < 0.01).

The Alphahold2-predicted structure of TDE_0127 is shown in Fig. 1A. A BLAST search for TrtA showed that amino acid residues 1–80 (out of 104 amino acids shown in rectangle in Fig. 1A) had similarity to the xenobiotic response element (XRE)-family helix-turn-helix DNA-binding domain-containing transcriptional regulator (Clusters of Orthologous Groups [COG] 1396 in the NCBI-curated protein classification resource). The function of the region of amino acid residues after 81 could not be predicted because the region did not show similarity to any other sequences of functional proteins. The amino acid sequence, with high identity, was detected in *T. denticola*-related species, including *Treponema phagedenis* (51%), *Treponema putidum* (82%), and *Treponema pedis* (64%). Interestingly, the deduced amino acid sequence from the XRE family transcriptional regulator (locus tag: EXT53_22335) in the Ec-140 genome of *Pectobacterium polaris*, which belongs to the order Enterobacterales, showed a 100% similarity with TrtA. After exposure of *T. denticola* ATCC 35405 to oxygen stress for 1 h, the expression of *trtA* was approximately four times higher than that under anaerobic conditions (Fig. 1B).

### Construction of the *trt*A-deletion mutant

The replacement of *trt*A with the *ermFermAM* cassette was confirmed using PCR and sequencing of the flanking region of the cassette (data not shown). The obtained mutant was designated *T. denticola* YK7. No morphological difference was detected between the mutant and wild-type strains. The growth rate of the wild-type and YK7 strain was similar, except at day 3 (Fig. 1C).

### Gene expression profile of the *trtA-*deletion mutant

**Fig 2:**
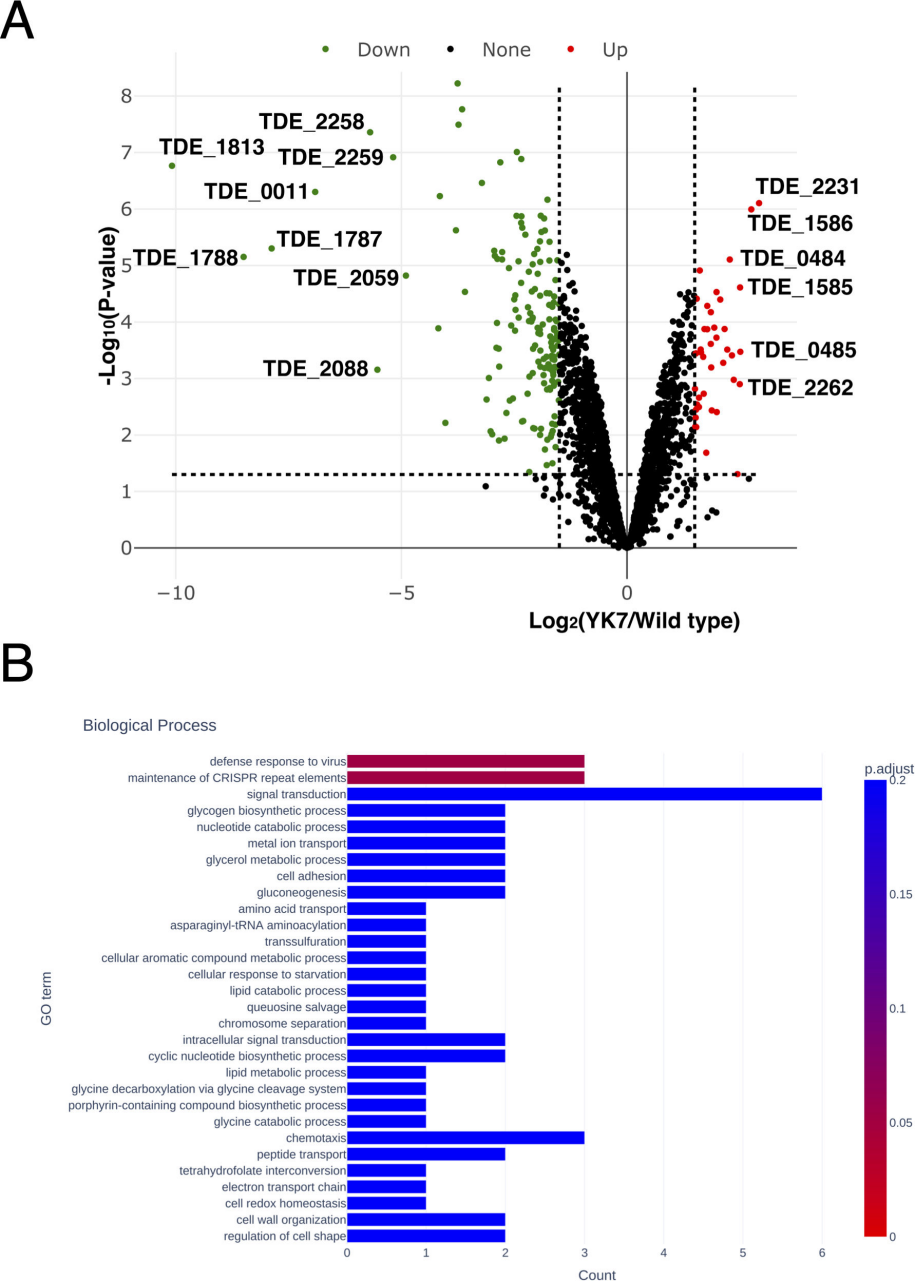
Gene expression profiles of the *trtA*-deletion mutant. (**A**) Volcano plots showing differentially expressed genes (*P* < 0.05). Horizontal dotted lines show the *P*-value cut-off of 0.05, and vertical dotted lines show the expression level log_2_ (YK7/wild-type) cut-off of <−1.5 or > 1.5. Red dots indicate upregulated genes, and green dots indicate downregulated genes. (**B**) GO enrichment of differentially expressed genes. Gene count from GO in the biological process. The gradient from red to blue on the bar indicates the adjusted *P*-value.

The comparison of gene expression between the wild-type strain and *trtA-*deletion mutant is shown in a volcano plot (Fig. 2A). The magnitude and range of difference in expression were larger for the downregulated genes (mean: 2^−2.37^-fold, range: 2^−1.5^ to 2^−10^-fold) than for the upregulated genes (mean: 2^1.92^-fold, range: 2^1.5^ to 2^2.9^-fold). Gene Ontology (GO) enrichment analysis of the differentially expressed genes revealed significant changes in biological process terms, specifically in the defense response to viruses and maintenance of Clustered Regularly Interspaced Short Palindromic (CRISPR) repeat elements (*P* < 0.05, Fig. 2B).

In total, 191 genes were significantly differentially expressed, and the number of downregulated genes was higher than that of upregulated genes (Table S1, *P* < 0.05, and false discovery rate [FDR] < 0.05, mutant/wild type <2^−1.5^ or >2^1.5^). The top 29 downregulated genes among the 153 identified genes are listed in Table 1. The downregulated genes in the YK7 strain included genes annotated to encode for peroxiredoxin, surface antigen BspA, 11 ABC transporter-associated proteins, 3 transcriptional regulators, 2 CRISPR-associated proteins, and 4 methyl-accepting chemotaxis proteins (Table 1; Table S1). The amino acid sequence encoded by TDE_0011 exhibited similarity with peroxiredoxin (COG0450) in residues 8–202 out of 215 residues, with an e-value of 2.53e-101. Among the 191 differentially expressed genes, TDE_0011 was the only gene that encodes a protein potentially directly involved in the oxygen response. The genes were annotated as encoding transcriptional regulators, including a C4-zinc finger domain protein; a DksA/TraR family protein (TDE_2088), which is a critical component of the rRNA transcription initiation machinery; an AbrB family transcriptional regulator (TDE_0344); and an ArsR family transcriptional regulator (TDE_1382).

**TABLE 1:** Genes downregulated in YK7

Gene	Annotation	Gene expression^*a*^ log_2_(YK7/wild type)
TDE_1813	Hypothetical protein	−10.08
TDE_1788	Conserved hypothetical protein	−8.50
TDE_1787	Conserved hypothetical protein	−7.88
TDE_0011	Alkyl hydroperoxide reductase/peroxiredoxin	−6.91
TDE_2258	Surface antigen BspA	−5.69
TDE_2088	C4 zinc finger domain protein, TraR/DksA family transcriptional regulator	−5.53
TDE_2259	Hypothetical protein	−5.18
TDE_2059	Hypothetical protein	−4.90
TDE_2057	Hypothetical protein	−4.18
TDE_2640	Hypothetical protein	−4.15
TDE_0742	Hypothetical protein	−4.03
TDE_1945	Hypothetical protein	−3.79
TDE_2058	Conserved hypothetical protein	−3.75
TDE_2055	Hemin-binding protein B	−3.73
TDE_2054	Conserved hypothetical protein	−3.65
TDE_0850	Methyl-accepting chemotaxis protein	−3.59
TDE_2056	Outer membrane hemin-binding protein A	−3.22
TDE_2715	Hypothetical protein	−3.11
TDE_0946	Hypothetical protein	−3.06
TDE_2141	Hypothetical protein	−3.02
TDE_1525	Hypothetical protein	−2.99
TDE_0012	Carbon starvation protein CstA, putative	−2.94
TDE_0969	Conserved hypothetical protein	−2.93
TDE_2303	Conserved hypothetical protein, putative endonuclease	−2.89
TDE_0741	Hypothetical protein	−2.88
TDE_0328	CRISPR-associated protein Cas1	−2.87
TDE_2301	FlhB domain-containing protein	−2.85
TDE_1526	Hypothetical protein	−2.84
TDE_2366	High-affinity branched-chain amino acid ABC transporter, permease protein	−2.83

^*a*^ *P* < 0.05 and false discovery rate (FDR) < 0.05.

BLAST analysis revealed that the N-terminal residues 1–122 encoded by TDE_2259 showed similarity with the HU family DNA-binding protein (Pfam: PF14848), as annotated in the Pfam database (2021) (26), whereas the C-terminal residues 130–227 showed similarity with the C-terminal domain of uncharacterized bacterial proteins (Pfam: PF14734). TDE_0874 encoded a sequence similar to that of segregation and condensation complex subunit ScpB, which is involved in chromosome segregation, transcription control, and DNA replication (27). Surface antigen BspA (encoded by TDE_2258, *lrrA*) has a leucine-rich repeat and is involved in adhering to Hep-2 cells and coaggregating with *T. forsythia* (28). The expression of the gene encoding the protease complex-associated polypeptide dentilisin was also decreased.

The top 30 of the 38 upregulated genes in the mutant are listed in Table 2. They included genes annotated as encoding internalin-related protein (TDE_2231), two ABC transporter-associated proteins, two methyl-accepting chemotaxis proteins, two peptidases, a cobalamin biosynthesis protein, and magnesium chelatase. Among the genes with decreased expression, TDE_0011 (encoding peroxiredoxin) is potentially involved in the oxygen stress response, and the expression of *trtA* was increased in a dentilisin-deficient mutant (25). Among the top five genes with increased expression, TDE_2231 (encoding an internalin-related protein) was the only gene whose expression level exceeded the average of that of all genes. We focused on TDE_0011 (encoding peroxiredoxin), dentilisin, and TDE_2231 (encoding internalin-related protein) for further analysis.

**TABLE 2:** Genes upregulated in YK7

Gene	Annotation	Gene expression^*a*^ log_2_(YK7/wild type)
TDE_2231	Internalin-related protein	2.93
TDE_1586	Conserved hypothetical protein	2.75
TDE_0485	Membrane protein, putative	2.51
TDE_1585	Conserved hypothetical protein	2.51
TDE_2262	Peptidase, U32 family	2.50
TDE_0755	Na+/H+ antiporter family protein	2.36
TDE_0413	Lipoprotein, putative	2.32
TDE_0484	Methyl-accepting chemotaxis protein	2.28
TDE_2525	Conserved hypothetical protein	2.22
TDE_0486	Membrane protein, putative	2.16
TDE_0338	Methyl-accepting chemotaxis protein-like protein	2.13
TDE_0299	Mutator mutT protein	2.07
TDE_0414	Hypothetical protein	1.99
TDE_0749	Cobalamin biosynthesis protein CobN, putative	1.98
TDE_1322	Hypothetical protein	1.98
TDE_2227	Membrane protein, putative	1.93
TDE_2620	Conserved hypothetical protein	1.88
TDE_2186	AMP-binding enzyme family protein K01897, long-chain acyl-CoA synthetase	1.87
TDE_0849	Membrane protein, putative	1.86
TDE_1489	Hypothetical protein	1.86
TDE_2226	ABC transporter, substrate-binding protein, putative	1.78
TDE_0706	Adenine-specific DNA modification methyltransferase	1.78
TDE_2471	Membrane protein, putative	1.72
TDE_0750	Magnesium chelatase, subunit D/I family	1.70
TDE_1336	Conserved hypothetical protein	1.69
TDE_0365	Conserved hypothetical protein	1.64
TDE_0546	Hypothetical protein	1.63
TDE_1915	Alcohol dehydrogenase, iron-containing	1.61
TDE_0364	ABC transporter, ATP-binding protein	1.59
TDE_1272	Oligopeptide/dipeptide ABC transporter, ATP-binding protein	1.59

^*a*^ *P* < 0.05 and false discovery rate (FDR) < 0.05.

### Effect of TrtA inactivation on oxygen stress response

**Fig 3:**
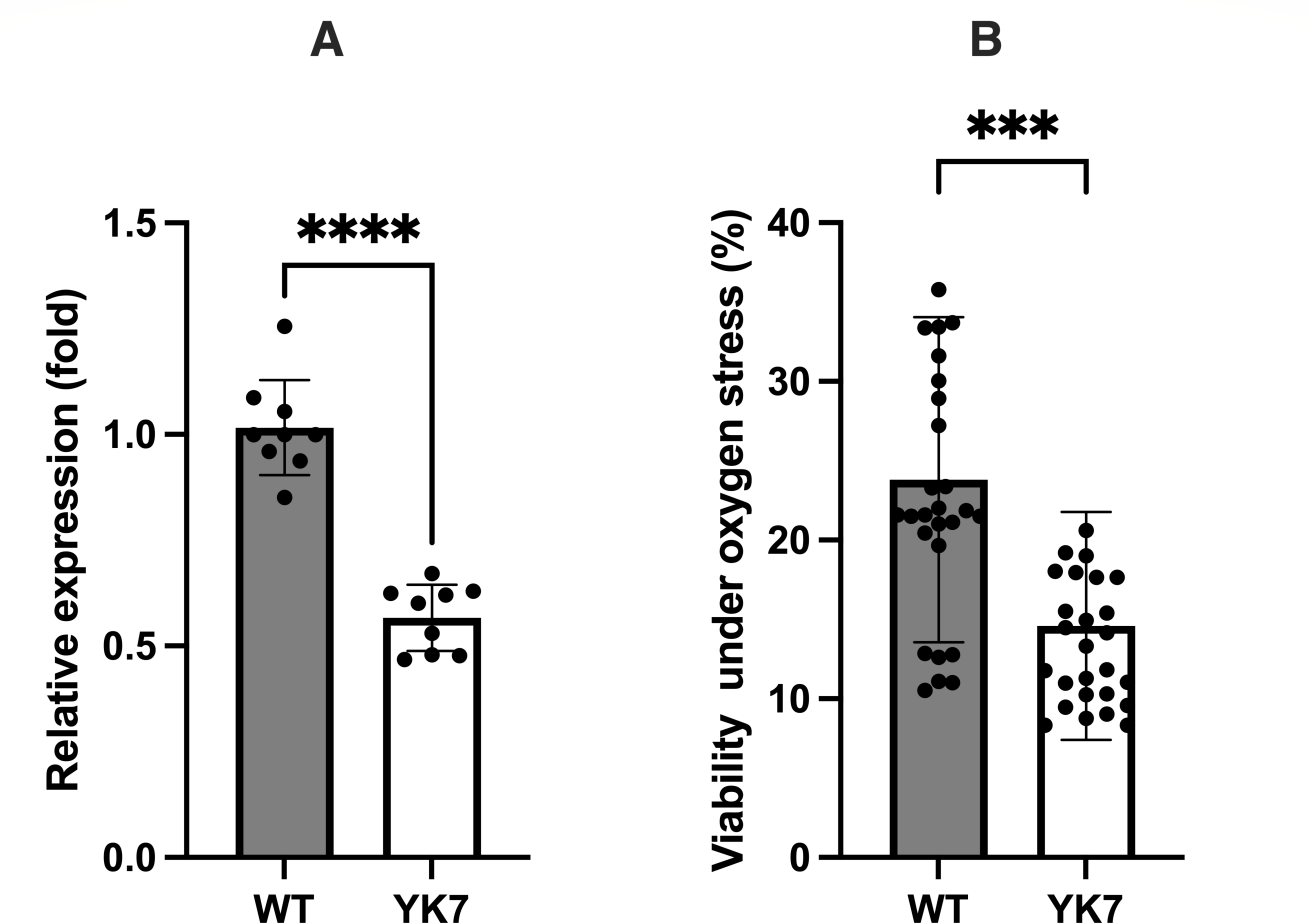
Expression of TDE_0011 (peroxiredoxin) and viability of the wild-type and YK7 strains under oxygen stress. (**A**) Expression of TDE_0011 (peroxiredoxin) in the wild-type and *trtA*-deletion mutant (YK7) strains under oxygen exposure for 1 h (*n* = 9). Statistically significant differences are indicated using asterisks (unpaired t-test, ****P < 0.0001). (**B**) The mean percentage of viability of the wild-type and *trtA*-deletion mutant (YK7) strains under oxygen exposure for 2 h. Viable cells were measured using BacTiter-Glo, and viability is expressed as the percentage of viable cells of the wild-type *T. denticola* strain cultured under anaerobic conditions for 2 h. Data are presented as mean ± standard deviation (*n* = 27), and statistically significant differences are indicated using asterisks (unpaired t-test, ***P < 0.001).

Among the downregulated genes in *T. denticola* YK7, the potential antioxidant enzyme-encoding gene, TDE_0011 (encoding peroxiredoxin), was significantly downregulated (Table 1). After exposure to oxygen, the expression of peroxiredoxin in the YK7 strain was significantly lower than that in the wild-type strain (Fig. 3A), indicating that TrtA is involved in peroxiredoxin expression. The viability of the *trtA*-deficient mutant was also significantly lower than that of the wild-type strain after oxygen exposure (Fig. 3B). These results suggested that TrtA is involved in the oxygen stress response of *T. denticola* via inducing peroxiredoxin expression. To confirm its regulation of peroxiredoxin, TrtA was expressed using plasmid pCF0127. However, the system did not function as expected because peroxiredoxin expression was increased by the transformation with the control plasmid pCF693Syn, which did not contain *trtA* (Fig. S1). To investigate the involvement of unexpected mutations in this phenomenon, mutations in the wild-type strain and the *trtA*-deletion mutant were investigated. A total of 101 common discrepancies were found in both strains (Table S2). In the wild-type strain, 207 discrepancies were detected, with 170 located within open reading frames. This number is similar to that of a previous report (29). In the mutant, 319 discrepancies were detected, with 263 located within open reading frames. Among them, 132 mutations occurred after the inactivation of TrtA. In a previous study, an increase in spontaneous mutation rate in the alkyl hydroperoxide reductase mutant was reported in *Bacteroides fragilis* and *Helicobacter pylori* (30, 31). It is possible that the decrease in peroxiredoxin due to the deletion of TrtA is involved in these mutations. Although the mutant showed a higher number of mutations than the wild-type strain, no mutations possibly affecting the regulation of peroxiredoxin (TDE_0011) were detected.

### Effect of TrtA on dentilisin expression

**Fig 4:**
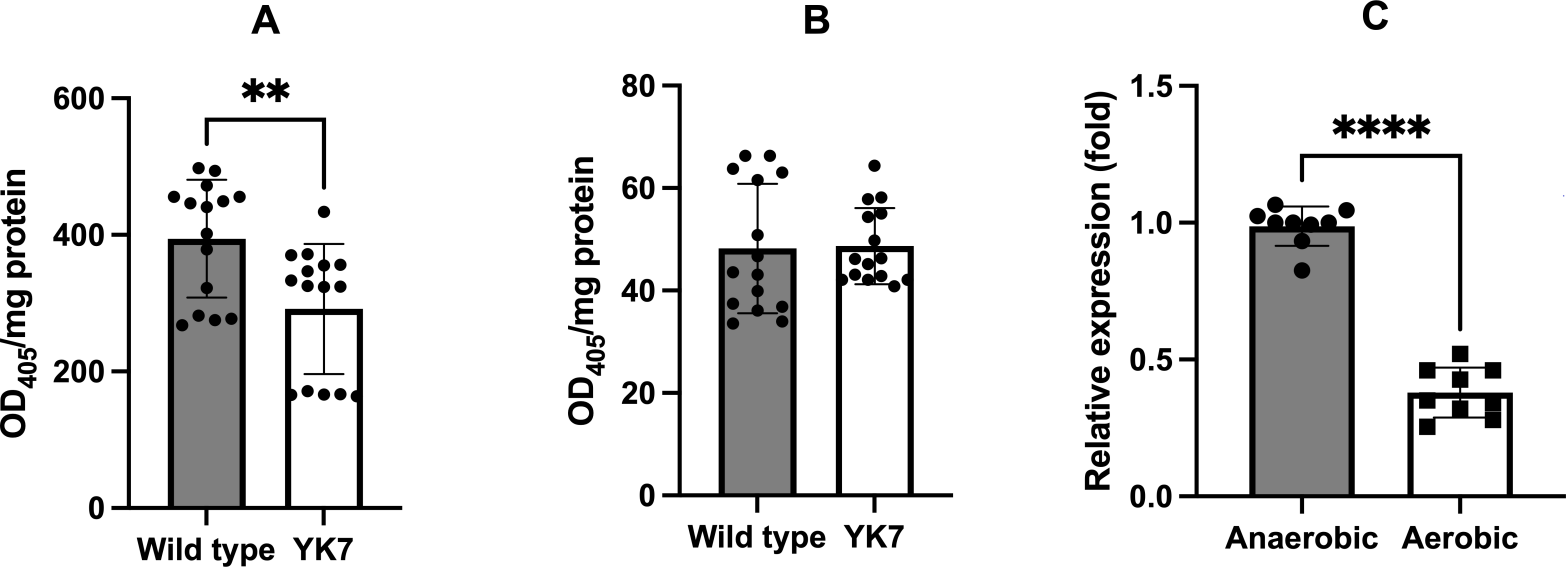
Effects of *trtA* inactivation and oxygen stress on proteolytic activity in *T. denticola.* The proteolytic activity of the *trtA*-deletion mutant (YK7) was compared with that of the wild-type strain. Dentilisin and arginine-specific oligopeptidase activities were measured using SAAPNA for dentilisin (**A**) and BAPNA for arginine-specific oligopeptidase (**B**) as a substrate; these are typical substrates for chymotrypsin and trypsin, respectively. After incubation at 37°C for 30 min, *p*-nitroaniline release was determined by measuring OD_405_. Data are presented as mean ± standard deviation (*n* = 15), and statistically significant differences are indicated using asterisks (unpaired t-test, **P < 0.01). The expression of dentilisin in the wild-type strain cultured under anaerobic and aerobic conditions for 1 h was evaluated using qRT-PCR (**C**). Data are presented as mean ± standard deviation (*n* = 9), and statistically significant differences are indicated using asterisks (unpaired t-test, ****P < 0.0001).

In the *trtA*-deletion mutant, the expression of protease-complex-associated polypeptide (encoded by TDE_0761), which forms a complex with dentilisin (encoded by TDE_0762), was decreased. In our previous study, the expression of *trtA* increased in the dentilisin-deficient mutant (25). In the YK7 strain, dentilisin activity was approximately 30% lower than that in the wild-type strain (Fig. 4A), whereas the activity of arginine-specific oligopeptidase (encoded by TDE_2140) in the mutant was not affected (Fig. 4B). These data indicated a decreased expression of the dentilisin gene cluster in the *trtA*-deletion mutant. To confirm the association between oxygen response and dentilisin expression, the expression of dentilisin under oxygen stress was evaluated in the wild-type strain. Dentilisin expression was significantly decreased under exposure to oxygen (Fig. 4C).

### Effect of TrtA on expression of internalin-related protein

**Fig 5:**
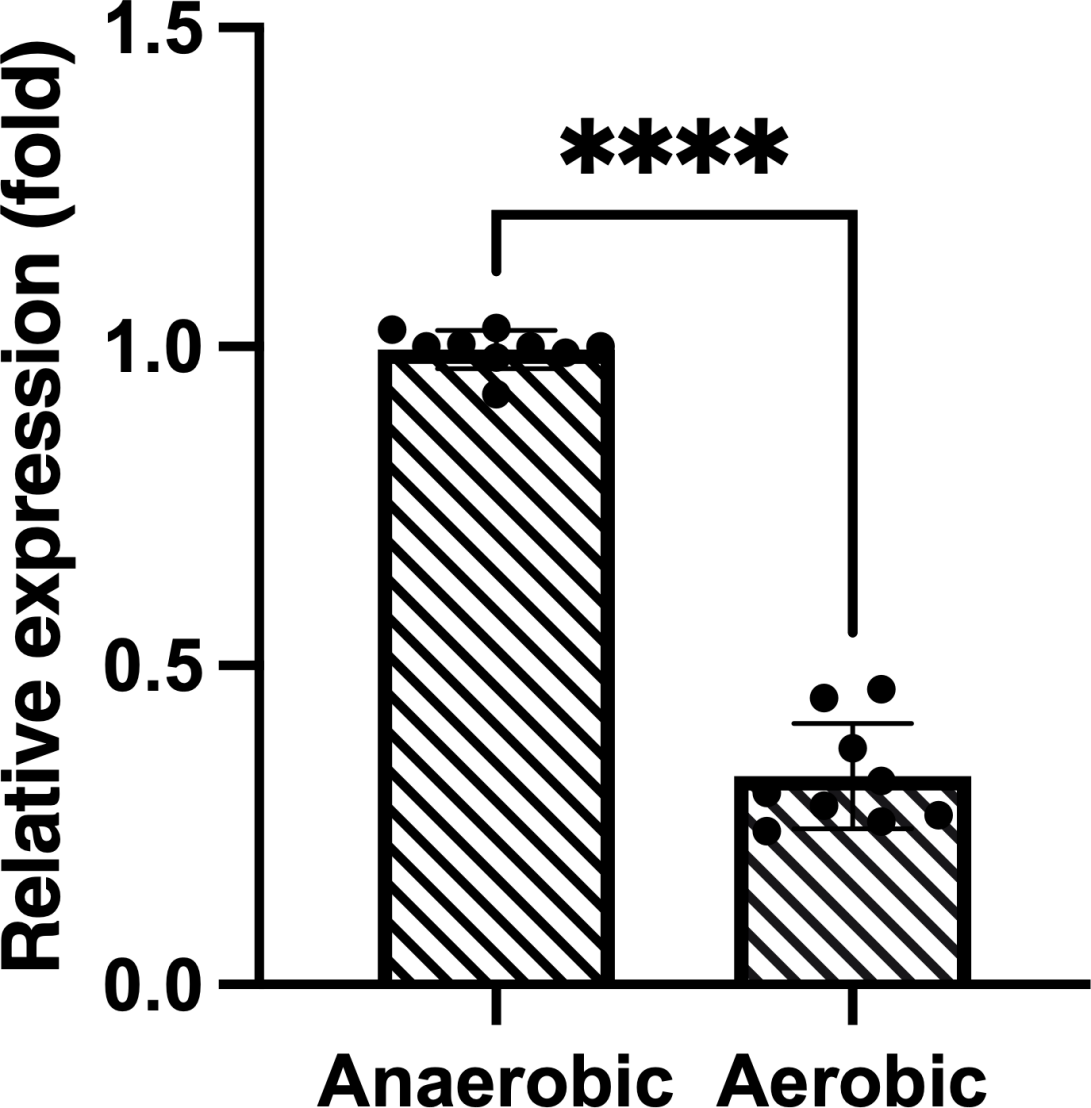
Expression of TDE_2231 in wild-type *T. denticola* under anaerobic and aerobic conditions. The expression of TDE_2231 in the wild-type strain was measured under anaerobic conditions and after 1 h of oxygen exposure (*n* = 9). Statistically significant differences are indicated using asterisks (unpaired t-test, ****P < 0.0001).

Internalin-related protein (TDE_2231) expression increased in the *trtA*-deletion mutant. The expression of TDE_2231 in the wild-type strain decreased under oxygen exposure (Fig. 5). This decrease was in line with the increase in TrtA expression in the wild-type strain under exposure to oxygen (Fig. 1B), suggesting that TrtA regulates TDE_2231.

**Fig 6:**
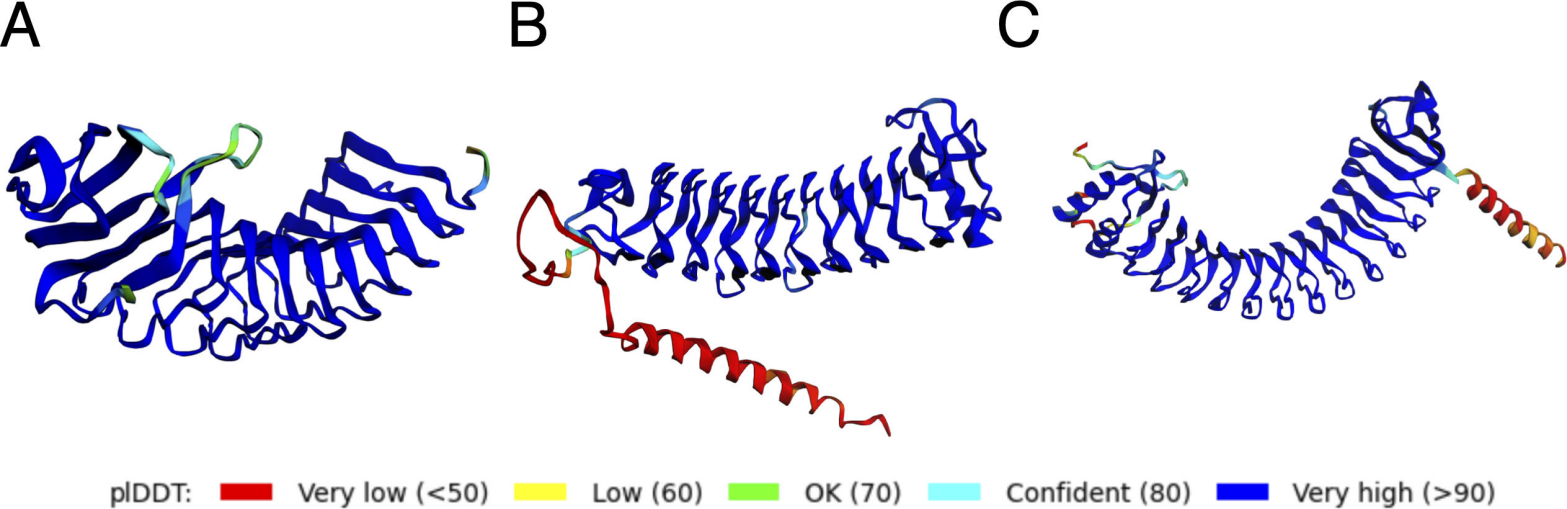
Predicted structure of internalin-related protein. Predicted structures of TDE_2231 (**A**), TDE_2258 (**B**), and TDE_2003 (**C**) via AlphaFold2. pLDDT: Predicted local distance difference test matching score.

TDE_2231 was annotated as encoding an internalin-related protein; however, its function remains unclear. The amino acid sequence of TDE_2231 shares similarity with the leucine-rich repeat domain (COG4886) at residues 86–273. The Alphahold2-predicted structure of TDE_2231 is shown in Fig. 6A; the structure was a classical curved solenoid. Foldseek analysis of the Protein Data Bank revealed that internalin A of *Listeria monocytogenes* has a similar structure to that of this protein (32). In addition to TDE_2231, the predicted structures of surface antigen BspA and TDE_2003, whose expression decreased in the *trtA-*deletion mutant, were of the classical curved-solenoid type (Fig. 6B and C).

**Fig 7:**
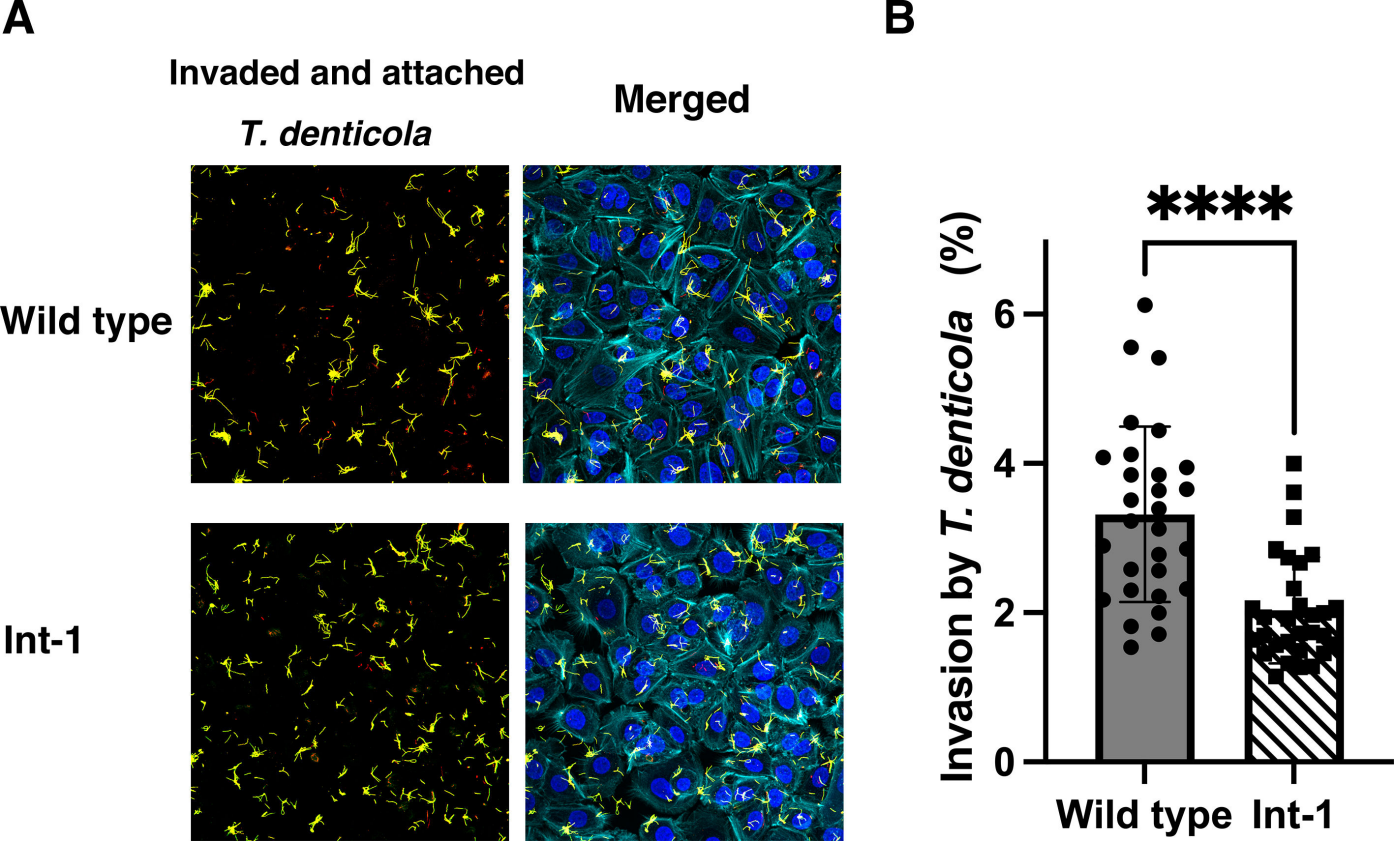
Intracellular invasion by *T. denticola* wild-type and TDE_2231-deletion strains. (**A**) TIGK cells were infected with *T. denticola* wild-type and TDE_2231-deletion (Int-1) strains at a multiplicity of infection of 100 and incubated for 24 h. The invaded and adherent *T. denticola* were observed using a confocal laser-scanning microscope. Invasion of *T. denticola* into TIGK cells was visualized using confocal laser-scanning microscopy following dual labeling. Intracellular *T. denticola* were stained red, extracellular *T. denticola* were stained yellow, and nuclei and actin of host cells were stained blue and cyan, respectively. (**B**) Invasion rate of *T. denticola* (%) was calculated using the following equation: *T. denticola* cells inside TIGK cells/(*T. denticola* cells inside + attached to TIGK cells) ×100. *T. denticola* cells were counted in 30 areas. Statistically significant differences are indicated using asterisks (unpaired t-test, ****P < 0.0001).

As internalin A is involved in adherence to host cells and the intracellular invasion of *L. monocytogenes*, a TDE_2231-deletion mutant (Int-1) was constructed to clarify the role of this protein in invasion by *T. denticola*. Attached and invaded *T. denticola* are shown in Fig. 7A. No difference in the arrangement of actin between the wild-type strain-infected cells and Int-1-infected cells was observed. The ratio of invasion (*T. denticola* cells inside telomerase-immortalized gingival keratinocyte [TIGK] cells/[*T. denticola* cells inside TIGK cells and *T. denticola* cells attached to TIGK cells] ×100) in the Int-1 strain was significantly lower than that in the wild-type strain (Fig. 7B), suggesting that TDE_2231 is associated with host cell invasion by *T. denticola*.

## DISCUSSION

*T. denticola* can tolerate oxygen but only at low levels (33), and the response mechanisms to oxygen stress have yet to be fully clarified. In the present study, the expression of the transcriptional regulator *trtA* increased in response to oxygen exposure. In the *trtA*-deletion mutant, 153 genes were downregulated, including those encoding peroxiredoxin and dentilisin, and 38 genes were upregulated, including those encoding internalin-related protein. The *trtA*-deletion mutant was more affected by oxygen stress than the wild-type strain.

The observed increase in TrtA expression under aerobic conditions was consistent with a previous study (24), indicating that TrtA participates in the response to oxygen stress in *T. denticola*. Based on the genome sequence of *T. denticola,* Nox (TDE_0096), peroxiredoxin (TDE_0011), and desulfoferrodoxin (TDE_1754) are predicted to play a role in the response of *T. denticola* to oxygen stress (20). Among the differentially expressed genes in the *trtA*-deletion mutant, the expression of the gene encoding peroxiredoxin was decreased. Under aerobic conditions, the expression of peroxiredoxin in the *trtA*-deletion mutant was approximately 55.8% that of the wild-type strain (Fig. 3A). These results indicated that peroxiredoxin expression was partially regulated by TrtA. In other anaerobes in subgingival crevices, such as *T. forsythia* and *P. intermedia*, proteins involved in the oxygen response, including superoxide dismutase and peroxiredoxin, are regulated by the regulator of antioxidant genes, OxyR (17–19). However, such a regulator-encoding gene was not detected in *T. denticola*.

In our previous study, OxtR1 participated in the oxygen stress response by regulating the expression of potential ferredoxins (23). In the OxtR1-deficient mutant, *trtA* expression was slightly increased, whereas the expression of Nox (TDE_0096), peroxiredoxin (TDE_0011), and desulfoferrodoxin (TDE_1754) remained unchanged, indicating that OxtR1 is not a master regulator of the oxygen stress response. After exposure to aerobic conditions, the proportion of viable cells of the TrtA-deletion mutant was significantly lower than that of the wild-type strain (Fig. 3B), suggesting that the decrease in peroxiredoxin expression affected oxygen stress resistance. In the *oxyR*-deficient mutant of *P. intermedia,* peroxiredoxin expression was completely abolished under aerobic conditions, whereas it was approximately half that of the wild-type strain under the same conditions. The difference suggested that peroxiredoxin was not regulated by the master regulator but by a dual regulator, including TrtA, in *T. denticola*. These results indicated that TrtA is a unique regulator involved in the response of *T. denticola* to oxygen stress.

Among the downregulated genes in the *trtA*-deletion mutant, three genes were annotated as encoding transcriptional regulators (TraR/DksA, AbrB, and ArsR families). In contrast, no transcriptional regulator was identified among the upregulated genes. The expression of the ArsR family transcriptional regulator increases in response to oxygen exposure (24), suggesting that it acts in collaboration with TrtA. The AbrB transcriptional regulator is involved in regulating the transition from a planktonic state to a biofilm in *Bacillus subtilis* (34). This transition state regulator regulates a large number of genes by binding to DNA at more than 600 sites (35, 36). The N-terminal domain of TDE_2259 is similar to the HU family DNA-binding protein, which is involved in regulating gene expression related to stress responses (37). This protein binds to DNA in a non-specific manner and is involved in *rpoS* translation, which induces the required genes under stress conditions (38). The expression of sigma factor 70 (*rpoS*), which regulates enzymes involved in oxidative stress responses, is induced by this protein (39). DksA is involved in the response to nutrient starvation conditions, along with (p)ppGpp (40). This gene influences the expression of an array of (p)ppGpp-dependent genes (41) and regulates the expression of virulence-associated lipoprotein OspC via post-transcriptional regulation of RpoS in *Borrelia burgdorferi* (42). Some of the numerous differentially expressed genes may be regulated by these downregulated potential transcriptional regulators. However, most of them seemed to exert secondary effects. In a previous study, the expression of many genes decreased in a *troR-*deletion mutant (43). Further analysis is required to distinguish the genes under the control of TrtA from those whose expression was decreased by a secondary effect.

A decrease in dentilisin expression was detected in the mutant strain under anaerobic conditions. We previously showed that TrtA was upregulated in a dentilisin-deficient mutant (25). However, the expression of dentilisin decreased in the wild-type strain under aerobic conditions, suggesting that dentilisin is not controlled by TrtA. A previous report suggested a link between dentilisin and iron uptake and homeostasis (44), and the hydrolysis of transferrin by this protease has been reported (45). Dentilisin may be involved in iron acquisition from serum transferrin. Control of cellular iron is essential to prevent oxygen stress, as overaccumulation of iron leads to the formation of reactive oxygen species through the Fenton reaction (46). Iron ion efflux is required for the response to oxygen stress (47). The changes in dentilisin expression in *trtA*-deletion mutants may result from crosstalk between the oxygen stress response and regulation of iron concentration in *T. denticola*. The expression of TDE_0749 (encoding a cobalamin biosynthesis protein, CobN) and TDE_0750 (encoding a magnesium chelatase) was also increased in the *trtA*-deletion mutant. In *P. gingivalis*, the heme uptake (Hmu) operon, involved in hemin uptake, contains *hmuS*, which encodes a CobN/magnesium chelatase fusion protein, and permeases (HmuT and HmuU) (48). The *hmuSTUV* genes are involved in transporting hemin into the cell from the periplasmic space (49). In the *T. denticola* genome, the orientation of the *cobN* and magnesium chelatase genes is opposite. Downstream of *cobN,* the genes are annotated as encoding the iron compound ABC transporter periplasmic iron compound-binding protein (TDE_0748), the iron compound ABC transporter permease protein (TDE_0747), and the iron compound ABC transporter, ATP-binding protein (TDE_0746). Although these genes were not upregulated in this study, this gene cluster may be involved in iron uptake from hemin. Further analysis, including the control of iron concentration, is required to clarify this regulatory network.

Internalin-related protein (encoded by TDE_2231) expression was increased in the *trtA*-deletion mutant. The expression of TDE_2231 decreased in the wild-type strain under exposure to oxygen. The upregulation of *trtA* induced this decrease in response to oxygen stress. These results suggested that TrtA regulated the increase in TDE_2231 expression. The structure of the protein encoded by TDE_2231 has similarity with that of internalin A in *L. monocytogenes*. Internalin A is involved in the adherence of *L. monocytogenes* to E-cadherin and host cell invasion by *L. monocytogenes* (50). Previous reports showed that *T. denticola* can invade epithelial cells (13, 51). Internalin-like proteins are involved in host cell invasion in other periodontopathic bacteria (52, 53). In the present study, the degree of invasion into TIGK cells in the *T. denticola* TDE_2231-deletion mutant (Int-1) was lower than that in the wild-type strain, indicating that this protein is involved in the interaction between *T. denticola* and host cells. However, the mechanism remains unclear. In this study, the response in the host cell, such as the arrangement of actin filaments, was not different between the wild-type-infected and the Int-1-deletion mutant-infected cells. Further analysis is required to elucidate the mechanisms underlying the altered intracellular invasion capacity observed in the mutant.

Surface antigen BspA and TDE_2003 also showed similarity with internalin, and their expression decreased in the *trtA*-deletion mutant. These proteins, with a classical curved-solenoid structure, may play a similar role in cell adhesion and invasion. Further analysis is required to investigate the function of each protein and its regulation.

A shuttle plasmid was used for the expression of *trtA* via transfection. However, peroxiredoxin expression was increased by transformation with the control plasmid pCF693Syn, but the increase in pCF0127 was not significant. The increase in target gene expression observed with the control plasmid was not seen in previous transfection experiments (23, 54). In this study, the expression of TDE_2259, which is similar to the HU family DNA-binding protein, decreased in the *trtA*-deletion mutant. This protein can bind to DNA in a non-specific manner and is involved in maintaining DNA structure (39, 55) and regulating gene expression (37, 56). In the *trtA*-deletion mutant, the expression of TDE_0011 was not eliminated. Possibly, the quantity of the HU family DNA-binding protein bound to the genomic DNA was changed by its binding to the control plasmid after transfection. This change in the quantity of the protein may affect the regulation of TDE_0011 expression in *T. denticola*, although further analysis of the regulation by the protein in this microorganism is required.

GO analysis revealed a significant downregulation of genes related to the maintenance of CRISPR repeat elements in the mutant compared with those in the wild type. In *Streptococcus mutans*, a *cas9*-deficient mutant exhibited impaired ability to resist stress induced by H_2_O_2_ (57). CRISPR-related genes may partially contribute to the response to oxygen stress.

There are several limitations to this study. Oxygen stress response plays an important role in the colonization of periodontopathic bacteria in the oral cavity (58, 59). In this study, we showed that increased TrtA expression under oxygen stress enhances the expression of peroxiredoxin (TDE_0011); however, the regulation mechanism of TrtA under oxygen stress has yet to be clarified. Although TrtA has a potential DNA-binding site, its binding to the upstream region of peroxiredoxin remains unconfirmed. The expression of peroxiredoxin was not completely abolished by inactivation of *trtA*, indicating that other molecules besides TrtA are also involved in the regulation of peroxiredoxin expression. Further analysis of TrtA expression regulation by oxygen stress and the regulation mechanism of peroxiredoxin expression is required to understand the complete regulation system of peroxiredoxin under oxygen stress.

In conclusion, the transcriptional regulator TrtA is involved in the oxygen stress response of *T. denticola*, possibly by regulating peroxiredoxin expression. Internalin-related protein is also controlled by TrtA. Dentilisin was not involved in the response to oxygen stress, although its expression decreased in the *trtA*-deletion mutant. This regulatory role of TrtA may shed light on *T. denticola* colonization during dysbiosis and its virulence.

## MATERIALS AND METHODS

### Bacterial strains and growth conditions

The bacterial strains and plasmids used in this study are listed in Table S3. *T. denticola* ATCC 35405, lacking the phage-derived region (*T. denticola* ATCC 35405 P(−)), was used as the wild-type strain. *T. denticola* strains were cultured in TYGVS medium (60) at 37 ℃ under anaerobic conditions (80% N_2_, 10% H_2_, and 10% CO_2_). For selection and culture of the mutant strain of *T. denticola*, 40 µg/mL erythromycin was added to the medium. *T. denticola* harboring pCF693Syn or pCF0127 was grown in TYGVS medium containing 10 µg/mL thiamphenicol. *Escherichia coli* strains were grown at 37°C in Luria–Bertani (LB) broth (Fujifilm Wako Chemicals, Tokyo, Japan) or on LB agar plates (Fujifilm Wako Chemicals) under aerobic conditions, and when appropriate, chloramphenicol (30 µg/mL) and erythromycin (300 µg/mL) were added to the medium to select *E. coli* harboring the plasmid. Primers used in this study are listed in Table S4.

### Construction of the mutants from *T. denticola* ATCC 35405

To investigate the role of TrtA and TDE_2231 in *T. denticola*, mutant strains were constructed. Plasmid pMCL191 was amplified via PCR using KOD-Plus-Neo (TOYOBO, Osaka, Japan) and the synthetic oligonucleotide primers pMCL191FW and PMCL191RV. To construct the *trtA*-deletion mutant, the *trtA* gene was replaced with *ermFermAM* (61). The sequence of *trtA,* as well as 1,030 bp upstream and 1,030 bp downstream, was amplified via PCR using the genomic DNA of *T. denticola* ATCC 35405 P(-) with the primer pair tde0127D and tde0127U. The fragment was ligated into pMCL191 using the In-Fusion HD Cloning Kit (Takara Bio, Kusatsu, Japan). The 5′ and 3′ flanking regions of *trtA* and pMCL191 were amplified from the obtained plasmid using the primers 127BF and 127BR. The erythromycin resistance cassette, *ermFermAM*, was amplified from pVA2198 using the primer pair EMF and EMR via PCR. The erythromycin cassette was ligated into the fragment obtained above using the In-Fusion HD Cloning Kit, and the resulting plasmid was introduced into *E. coli* TOP10.

For TDE_2231, a fragment containing *intT1* and 1,038 bp of the 5′ and 1,060 bp of the 3′ flanking regions of *intT1* was amplified via PCR using the primers 2231F and 2231R and ligated into pMCL191 using the In-Fusion HD Cloning Kit. The resulting plasmid was transformed into *E. coli* TOP10. The 5′ and 3′ flanking regions of *intT1* and pMCL191 were amplified from the obtained plasmid using the primers 2231VF and 2231VR, ligated with the *ermB* cassette amplified using primers ermB2231F and ermB2231R from pVA2198 using the In-Fusion HD Cloning Kit, and introduced into *E. coli* TOP10.

The obtained plasmids, pK127 (for the *trtA*-deletion mutant) and pK2231 (for the TDE_2231-deletion mutant), were linearized by digesting with *Bam*HI and *Eco*RI, respectively. Obtained fragments were introduced into *T. denticola* ATCC 35405 P(-) using the CaCl_2_ method as described previously (62). Briefly, late-logarithmic phase *T. denticola* cultures (100 mL) were harvested. The cells were washed four times with ice-cold buffer consisting of 15% glycerol containing 50 mM CaCl_2_ and then resuspended in 1 mL of ice-cold buffer. Thereafter, 80 µL of the cells was mixed with 10 µg of the digested plasmid. The mixture was incubated on ice for 10 min, at 50°C for 1 min, and on ice again for 5 min. The cells were immediately inoculated into 5 mL of TYGVS medium and incubated under anaerobic conditions for 2 days. After incubation, the cells were plated onto TYGVS medium containing 0.8% noble agar (Difco) with 40 µg/mL erythromycin and incubated anaerobically for 10 days. The resulting colonies were screened using PCR. The *trtA*-deletion mutant and TDE_2231 mutant were designated YK7 and Int-1, respectively.

### Evaluation of the growth rate of the mutant strain of *T. denticola*

*T. denticola* strains were grown for 2 days at 37°C on TYGVS broth. After reaching the early stationary phase, the culture was diluted with fresh TYGVS broth to an optical density at 660 nm (OD_660_) of 1.0. The diluted cultures (5 mL) were then incubated at 37°C under anaerobic conditions. Bacterial growth was monitored by measuring OD_660_ with a spectrophotometer (Mini Photo 518R; TAITEC, Tokyo, Japan) at predetermined time points.

### Viability of *T. denticola* strains under oxygen stress

Cultures of *T. denticola* (2 mL) were aliquoted into 12-well culture plates (Corning Incorporated, Kennebunk, ME, USA), and the cells were cultured under aerobic conditions with continuous shaking (500 rpm) or anaerobic conditions at 37°C for 2 h. The control for each strain was incubated under anaerobic conditions at 37°C for 2 h. After incubation, 1 mL aliquots were taken from each well and mixed with 1 mL of BacTiter-Glo (Promega Japan, Tokyo, Japan). 200 µL of the mixture was added to a 96-well black polystyrene plate (Corning), and chemiluminescence was measured using Spectramax M5e (Molecular Devices Japan, Tokyo, Japan).

### RNA sequencing of the *trtA*-deletion mutant

*T. denticola* ATCC 35405 P(-) and YK7, grown in TYGVS medium (OD_660_ = ∼0.4–0.6), were harvested. RNA was extracted using TRIzol Reagent (Invitrogen, Carlsbad, CA, USA), and residual DNA was removed using the TURBO DNA-free kit (Thermo Fisher Scientific, Waltham, MA, USA), as described previously (63). After confirming the quality of the RNA using Tape Station (Agilent Technologies, Tokyo, Japan), a library was constructed from the extracted RNA using the TruSeq Stranded mRNA Sample Prep kit (Illumina) according to the manufacturer’s instructions. The obtained library was sequenced on the HiSeq 2500 (Illumina); 100 bp paired-end reads were trimmed using cutadapt (https://cutadapt.readthedocs.org/en/stable/). Thereafter, the obtained sequences were mapped using Salmon (64) and the ASM818v1.gentrome of *T. denticola* ATCC 35405. The differentially expressed genes were identified using TCC in R (65), where findings with *P* < 0.05 and FDR < 0.05 were considered significant. Differentially expressed genes were subjected to GO analysis using GOATOOLS v. 1.1.6 (66).

### Bioinformatics analysis

Analysis of deduced amino acid sequences was carried out using BLAST (https://blast.ncbi.nlm.nih.gov/Blast.cgi), and the structures were predicted using AlphaFold 2/CollabFold (https://colab.research.google.com/github/sokrypton/ColabFold/blob/main/AlphaFold2.ipynb) (67–69).

### Evaluation of gene expression using qRT-PCR

To investigate the expression of the differentially expressed genes, qRT-PCR was performed. *T. denticola* cells grown in TYGVS medium (OD_660_ = ∼0.4-0.6) under anaerobic conditions were harvested. To evaluate the effect of oxygen exposure, cells (OD_660_ = ∼0.4-0.6) were harvested after being cultured under aerobic conditions with continuous shaking (500 rpm) for 1 h. Total RNA from *T. denticola* strains was isolated as described above. cDNA was synthesized using ReverTra Ace (Toyobo, Osaka, Japan), and 1 µL of the synthesized cDNA was added to 10 µL of TaqMan Fast Universal PCR Master Mix (Applied Biosystems), 1 µL gene-specific primers (Table S3), Taqman probe mix, and 8 µL RNase-free water in a well. qRT-PCR was carried out in triplicate using the StepOne Plus Real-time PCR system (Applied Biosystems). 16S rRNA was used as an internal control. The expression of each gene was evaluated using the relative standard curve method.

### Detection of the mutation in the genome of the wild-type strain and the *trtA-*deletion mutant of *T. denticola*

Genomic DNA was isolated from *T. denticola* ATCC 35405 P(−) and *T. denticola* YK7 as previously described (70). A sequence library was constructed from 100 ng of genomic DNA using Illumina DNA Prep (M) Tagmentation kit (Illumina) according to the manufacturer’s instructions. Library sequencing was carried out using the MiSeq (Illumina) platform (150 paired-end reads). After trimming the raw sequence using fastp v. 0.20.1 (71), the obtained sequence provided approximately 100-fold coverage of the genome sequence of *T. denticola* ATCC 35405 in GenBank (https://www.ncbi.nlm.nih.gov/datasets/genome/GCF_000008185.1). Variations in the genomic sequences were investigated using Breseq v. 0.35.4 (72).

### Effect of inactivation of TrtA on proteolytic activity

In addition to that of dentilisin, the activity of arginine-specific oligopeptidase (OpdB) was measured as a control. The measurement of proteolytic activity was performed as previously described (73). Briefly, *T. denticola* cells were collected when the OD_660_ reached approximately 0.6, and then the culture was adjusted to an OD_660_ of 0.5 with fresh TYGVS medium. *T. denticola* cells were washed and suspended in phosphate-buffered saline (PBS). A 5 mL aliquot of the suspension was mixed with 5 mL of 100 mM Tris-HCl buffer (pH 8.0). The protein concentration of the suspension was determined using the BCA Protein Assay Kit (Thermo Fisher Scientific). The samples (90 µL) were mixed with 10 µL of 1 mM N-succinyl-L-alanyl-L-alanyl-L-prolyl-L-phenylalanine *p*-nitroanilide (SAAPNA; Sigma Chemical Company, St. Louis, MO, USA) for dentilisin or N-Benzoyl-DL-arginine 4-nitroanilide hydrochloride (BAPNA; Sigma Chemical Company) for OpdB and incubated at 37°C for 30 min. The reaction was stopped by adding 100 µL of 20% acetic acid. *p*-nitroaniline release was determined by measuring the OD_405_ using a Spectramax M5e. Each proteolytic activity was normalized with the protein concentration.

### Assessment of intracellular invasion by *T. denticola*

TDE_2231 has a leucine-rich repeat and is annotated as an internalin-related protein. Internalin is involved in invasion by *L. monocytogenes* (32). To investigate the function of TDE_2231 in *T. denticola*, the adherence and invasion capacity of a TDE_2231-deficient mutant were investigated. TIGK cells (74) were cultured in keratinocyte growth medium (DermaLife basal medium, Lifeline Cell Technology, LLC, San Diego, CA, USA). Confluent cells were detached using a trypsin/EDTA solution, and the number of cells was adjusted to approximately 10^5^ /mL of cell culture medium in a 24-well flask. The cells were then sub-cultured for 3 days.

To observe both intracellular and extracellular *T. denticola* cells, a double-fluorescence technique was performed as described previously (75). Briefly, mid-log phase *T. denticola* strains were used to infect TIGK cells at a multiplicity of infection of 100, incubated for 24 h, washed with PBS, and treated with 4% neutral paraformaldehyde for 1 h. Samples were incubated with a rabbit anti-*T*. *denticola serum* (76) diluted 1:500 in Can Get Signal Immunostain Immunoreaction Enhancer Solution (Toyobo) for 60 min, followed by incubation with Alexa Fluor 488 anti-rabbit antibody (1:100, Thermo Fisher Scientific) to visualize the attached bacteria after washing with PBS. Samples were permeabilized with 0.1% Triton X-100 solution for 5 min, blocked with blocking solution (Invitrogen) for 30 min, and stained for intracellular *T. denticola* using rabbit anti-*T*. *denticola* serum followed by Alexa Fluor 568 anti-rabbit IgG (Invitrogen), diluted 1:100, as described above. Actin filaments were stained with Alexa Fluor 647 conjugated to phalloidin (Invitrogen) to visualize the cellular cytoskeleton, and cell nuclei were stained with 4′,6-diamidino-2-phenylindole (DAPI; Dojindo Laboratories, Tokyo, Japan) for 30 min according to the supplier’s recommendations.

Samples were observed using a confocal laser-scanning microscope (CLSM) (LSM 880, Carl Zeiss MicroImaging, Göttingen, Germany), and images were captured using ZEN software (Carl Zeiss). Scanned areas were randomly selected, and 30 areas were examined for each group. In the CLSM images, the number of red-colored intracellular bacteria, yellow-colored extracellular bacteria, and blue-colored host cells was counted, and the ratio of invasion (*T. denticola* cells inside TIGK cells/(*T. denticola* cells inside TIGK cells and *T. denticola* cells attached to TIGK cells) ×100) was calculated.

### Complementation of *trtA* via plasmid transfection

The backbone of pCF693Syn and the *ermB* promoter were amplified from pCF0259 using the primer pair pCF-F and ermBPRR_127, via PCR. *trtA* was amplified using PCR with the primer pair pCF_127F and pCF_127R and inserted downstream of the *ermB* promoter of the backbone of pCF693Syn using the In-Fusion HD Cloning Kit and introduced into *E. coli* TOP10. The obtained plasmid was designated as pCF0127. The sequence from the *ermB* promoter to TDE_0127 in pCF0127 was confirmed using Sanger sequencing. pCF0127 and pCF693Syn were introduced into dam^−^/dcm^−^ *E. coli*. The plasmids were introduced into *T. denticola* ATCC 35405 P(-) using electroporation as described previously (77). Transformants were screened using TYGVS agar plates containing 10 µg/mL thiamphenicol (54). Gene expression in the strain harboring these plasmids was evaluated using qRT-PCR as described above.

### Statistical analysis

Each experiment was performed independently in triplicate at least three times. Differences between groups were analyzed using an unpaired t-test using GraphPad Prism 9.5.1 (GraphPad Software, Boston, MA, USA). *P* values of 0.05 were considered significant*.*

## Data Availability

The raw data obtained using RNA sequencing analysis have been deposited in the Sequence Read Archive of the DNA Data Bank of Japan (DDBJ/DRA) (https://www.ddbj.nig.ac.jp/dra) under submission IDs PRJDB18475 and PRJDB35976. Other raw data will be made available on request.

## SUPPLEMENTAL MATERIAL

Supplemental material aem.00750-25-s0001.docx Tables S1 to S4; Fig. S1.

ASM does not own the copyrights to Supplemental Material that may be linked to, or accessed through, an article. The authors have granted ASM a non-exclusive, world-wide license to publish the Supplemental Material files. Please contact the corresponding author directly for reuse.

## References

[1] Pihlstrom BL, Michalowicz BS, Johnson NW. 2005. Periodontal diseases. Lancet 366:1809–1820. doi:10.1016/S0140-6736(05)67728-8 16298220

[2] Kinane DF, Stathopoulou PG, Papapanou PN. 2017. Periodontal diseases. Nat Rev Dis Primers 3:17038. doi:10.1038/nrdp.2017.38 28805207

[3] Paster BJ, Boches SK, Galvin JL, Ericson RE, Lau CN, Levanos VA, Sahasrabudhe A, Dewhirst FE. 2001. Bacterial diversity in human subgingival plaque. J Bacteriol 183:3770–3783. doi:10.1128/JB.183.12.3770-3783.2001 11371542 PMC95255

[4] Hajishengallis G, Lamont RJ. 2012. Beyond the red complex and into more complexity: the polymicrobial synergy and dysbiosis (PSD) model of periodontal disease etiology. Mol Oral Microbiol 27:409–419. doi:10.1111/j.2041-1014.2012.00663.x 23134607 PMC3653317

[5] Lamont RJ, Koo H, Hajishengallis G. 2018. The oral microbiota: dynamic communities and host interactions. Nat Rev Microbiol 16:745–759. doi:10.1038/s41579-018-0089-x 30301974 PMC6278837

[6] Moore WE, Holdeman LV, Cato EP, Smibert RM, Burmeister JA, Ranney RR. 1983. Bacteriology of moderate (chronic) periodontitis in mature adult humans. Infect Immun 42:510–515. doi:10.1128/iai.42.2.510-515.1983 6642641 PMC264458

[7] Socransky SS, Haffajee AD, Cugini MA, Smith C, Kent RL Jr. 1998. Microbial complexes in subgingival plaque. J Clinic Periodontology 25:134–144. doi:10.1111/j.1600-051X.1998.tb02419.x 9495612

[8] Griffen AL, Beall CJ, Campbell JH, Firestone ND, Kumar PS, Yang ZK, Podar M, Leys EJ. 2012. Distinct and complex bacterial profiles in human periodontitis and health revealed by 16S pyrosequencing. ISME J 6:1176–1185. doi:10.1038/ismej.2011.191 22170420 PMC3358035

[9] Chen T, Marsh PD, Al-Hebshi NN. 2022. SMDI: an index for measuring subgingival microbial dysbiosis. J Dent Res 101:331–338. doi:10.1177/00220345211035775 34428955 PMC8982011

[10] Ishihara K. 2010. Virulence factors of Treponema denticola. Periodontol 2000 54:117–135. doi:10.1111/j.1600-0757.2009.00345.x 20712637

[11] Miyamoto M, Ishihara K, Okuda K. 2006. The Treponema denticola surface protease dentilisin degrades interleukin-1 beta (IL-1 beta), IL-6, and tumor necrosis factor alpha. Infect Immun 74:2462–2467. doi:10.1128/IAI.74.4.2462-2467.2006 16552080 PMC1418930

[12] Yamazaki T, Miyamoto M, Yamada S, Okuda K, Ishihara K. 2006. Surface protease of Treponema denticola hydrolyzes C3 and influences function of polymorphonuclear leukocytes. Microbes Infect 8:1758–1763. doi:10.1016/j.micinf.2006.02.013 16815066

[13] Inagaki S, Kimizuka R, Kokubu E, Saito A, Ishihara K. 2016. Treponema denticola invasion into human gingival epithelial cells. Microb Pathog 94:104–111. doi:10.1016/j.micpath.2016.01.010 26806000

[14] Bamford CV, Fenno JC, Jenkinson HF, Dymock D. 2007. The chymotrypsin-like protease complex of Treponema denticola ATCC 35405 mediates fibrinogen adherence and degradation. Infect Immun 75:4364–4372. doi:10.1128/IAI.00258-07 17591786 PMC1951159

[15] Fu H, Yuan J, Gao H. 2015. Microbial oxidative stress response: novel insights from environmental facultative anaerobic bacteria. Arch Biochem Biophys 584:28–35. doi:10.1016/j.abb.2015.08.012 26319291

[16] Diaz PI, Slakeski N, Reynolds EC, Morona R, Rogers AH, Kolenbrander PE. 2006. Role of oxyR in the oral anaerobe Porphyromonas gingivalis. J Bacteriol 188:2454–2462. doi:10.1128/JB.188.7.2454-2462.2006 16547032 PMC1428421

[17] Honma K, Mishima E, Inagaki S, Sharma A. 2009. The OxyR homologue in Tannerella forsythia regulates expression of oxidative stress responses and biofilm formation. Microbiology (Reading) 155:1912–1922. doi:10.1099/mic.0.027920-0 19389765 PMC2782426

[18] Kikuchi Y, Okamoto-Shibayama K, Kokubu E, Ishihara K. 2021. OxyR inactivation reduces the growth rate and oxidative stress defense in Capnocytophaga ochracea. Anaerobe 72:102466. doi:10.1016/j.anaerobe.2021.102466 34673216

[19] Naito M, Belvin BR, Shoji M, Gui Q, Lewis JP. 2021. Insertional inactivation of Prevotella intermedia OxyR results in reduced survival with oxidative stress and in the presence of host cells. Microorganisms 9:551. doi:10.3390/microorganisms9030551 33800047 PMC7999485

[20] Seshadri R, Myers GSA, Tettelin H, Eisen JA, Heidelberg JF, Dodson RJ, Davidsen TM, DeBoy RT, Fouts DE, Haft DH, et al.. 2004. Comparison of the genome of the oral pathogen Treponema denticola with other spirochete genomes. Proc Natl Acad Sci USA 101:5646–5651. doi:10.1073/pnas.0307639101 15064399 PMC397461

[21] Frederick JR, Sarkar J, McDowell JV, Marconi RT. 2011. Molecular signaling mechanisms of the periopathogen, Treponema denticola. J Dent Res 90:1155–1163. doi:10.1177/0022034511402994 21447698 PMC3173007

[22] Bian J, Liu X, Cheng YQ, Li C. 2013. Inactivation of cyclic Di-GMP binding protein TDE0214 affects the motility, biofilm formation, and virulence of Treponema denticola*.* J Bacteriol 195:3897–3905. doi:10.1128/JB.00610-13 23794624 PMC3754597

[23] Numata Y, Kikuchi Y, Sato T, Okamoto-Shibayama K, Ando Y, Miyai-Murai Y, Kokubu E, Ishihara K. 2024. Novel transcriptional regulator OxtR1 regulates potential ferrodoxin in response to oxygen stress in Treponema denticola. Anaerobe 87:102852. doi:10.1016/j.anaerobe.2024.102852 38614291

[24] McHardy I, Keegan C, Sim JH, Shi W, Lux R. 2010. Transcriptional profiles of Treponema denticola in response to environmental conditions. PLoS One 5:e13655. doi:10.1371/journal.pone.0013655 21048920 PMC2965109

[25] Arai Y, Kikuchi Y, Okamoto-Shibayama K, Kokubu E, Shintani S, Ishihara K. 2020. Investigation of the potential regulator proteins associated with the expression of major surface protein and dentilisin in Treponema denticola. J Oral Microbiol 12:1829404. doi:10.1080/20002297.2020.1829404 33149843 PMC7586716

[26] Mistry J, Chuguransky S, Williams L, Qureshi M, Salazar GA, Sonnhammer ELL, Tosatto SCE, Paladin L, Raj S, Richardson LJ, Finn RD, Bateman A. 2021. Pfam: the protein families database in 2021. Nucleic Acids Res 49:D412–D419. doi:10.1093/nar/gkaa913 33125078 PMC7779014

[27] Hoencamp C, Rowland BD. 2023. Genome control by SMC complexes. Nat Rev Mol Cell Biol 24:633–650. doi:10.1038/s41580-023-00609-8 37231112

[28] Ikegami A, Honma K, Sharma A, Kuramitsu HK. 2004. Multiple functions of the leucine-rich repeat protein LrrA of Treponema denticola. Infect Immun 72:4619–4627. doi:10.1128/IAI.72.8.4619-4627.2004 15271922 PMC470683

[29] Yokogawa T, Nagano K, Fujita M, Miyakawa H, Iijima M. 2022. Characterization of a Treponema denticola ATCC 35405 mutant strain with mutation accumulation, including a lack of phage-derived genes. PLoS One 17:e0270198. doi:10.1371/journal.pone.0270198 35749516 PMC9231711

[30] Olczak AA, Olson JW, Maier RJ. 2002. Oxidative-stress resistance mutants of Helicobacter pylori. J Bacteriol 184:3186–3193. doi:10.1128/JB.184.12.3186-3193.2002 12029034 PMC135082

[31] Rocha ER, Smith CJ. 1999. Role of the alkyl hydroperoxide reductase (ahpCF) gene in oxidative stress defense of the obligate anaerobe Bacteroides fragilis. J Bacteriol 181:5701–5710. doi:10.1128/JB.181.18.5701-5710.1999 10482511 PMC94090

[32] Bonazzi M, Lecuit M, Cossart P. 2009. Listeria monocytogenes internalin and E-cadherin: from structure to pathogenesis. Cell Microbiol 11:693–702. doi:10.1111/j.1462-5822.2009.01293.x 19191787

[33] Caldwell CE, Marquis RE. 1999. Oxygen metabolism by Treponema denticola. Oral Microbiol Immunol 14:66–72. doi:10.1034/j.1399-302x.1999.140109.x 10204483

[34] Strauch MA, Hoch JA. 1993. Transition-state regulators: sentinels of Bacillus subtilis post-exponential gene expression. Mol Microbiol 7:337–342. doi:10.1111/j.1365-2958.1993.tb01125.x 8459762

[35] Chumsakul O, Takahashi H, Oshima T, Hishimoto T, Kanaya S, Ogasawara N, Ishikawa S. 2011. Genome-wide binding profiles of the Bacillus subtilis transition state regulator AbrB and its homolog Abh reveals their interactive role in transcriptional regulation. Nucleic Acids Res 39:414–428. doi:10.1093/nar/gkq780 20817675 PMC3025583

[36] Rösch TC, Oviedo-Bocanegra LM, Fritz G, Graumann PL. 2018. SMTracker: a tool for quantitative analysis, exploration and visualization of single-molecule tracking data reveals highly dynamic binding of B. subtilis global repressor AbrB throughout the genome. Sci Rep 8:15747. doi:10.1038/s41598-018-33842-9 30356068 PMC6200787

[37] Oberto J, Nabti S, Jooste V, Mignot H, Rouviere-Yaniv J. 2009. The HU regulon is composed of genes responding to anaerobiosis, acid stress, high osmolarity and SOS induction. PLoS One 4:e4367. doi:10.1371/journal.pone.0004367 19194530 PMC2634741

[38] Balandina A, Claret L, Hengge-Aronis R, Rouviere-Yaniv J. 2001. The Escherichia coli histone-like protein HU regulates rpoS translation. Mol Microbiol 39:1069–1079. doi:10.1046/j.1365-2958.2001.02305.x 11251825

[39] Balandina A, Kamashev D, Rouviere-Yaniv J. 2002. The bacterial histone-like protein HU specifically recognizes similar structures in all nucleic acids. DNA, RNA, and their hybrids. J Biol Chem 277:27622–27628. doi:10.1074/jbc.M201978200 12006568

[40] Gourse RL, Chen AY, Gopalkrishnan S, Sanchez-Vazquez P, Myers A, Ross W. 2018. Transcriptional responses to ppGpp and DksA. Annu Rev Microbiol 72:163–184. doi:10.1146/annurev-micro-090817-062444 30200857 PMC6586590

[41] Boyle WK, Groshong AM, Drecktrah D, Boylan JA, Gherardini FC, Blevins JS, Samuels DS, Bourret TJ. 2019. DksA controls the response of the lyme disease spirochete Borrelia burgdorferi to starvation. J Bacteriol 201:e00582-18. doi:10.1128/JB.00582-18 30478087 PMC6351744

[42] Mason C, Thompson C, Ouyang Z. 2020. DksA plays an essential role in regulating the virulence of Borrelia burgdorferi. Mol Microbiol 114:172–183. doi:10.1111/mmi.14504 32227372 PMC8331073

[43] Saraithong P, Goetting-Minesky MP, Durbin PM, Olson SW, Gherardini FC, Fenno JC. 2020. Roles of TroA and TroR in metalloregulated growth and gene expression in Treponema denticola. J Bacteriol 202:e00770-19. doi:10.1128/JB.00770-19 31932313 PMC7167467

[44] Goetting-Minesky MP, Godovikova V, Fenno JC. 2021. Approaches to understanding mechanisms of dentilisin protease complex expression in Treponema denticola. Front Cell Infect Microbiol 11:668287. doi:10.3389/fcimb.2021.668287 34084756 PMC8167434

[45] Uitto VJ, Grenier D, Chan EC, McBride BC. 1988. Isolation of a chymotrypsinlike enzyme from Treponema denticola. Infect Immun 56:2717–2722. doi:10.1128/iai.56.10.2717-2722.1988 3166451 PMC259634

[46] Touati D. 2000. Iron and oxidative stress in bacteria. Arch Biochem Biophys 373:1–6. doi:10.1006/abbi.1999.1518 10620317

[47] Bradley JM, Svistunenko DA, Wilson MT, Hemmings AM, Moore GR, Le Brun NE. 2020. Bacterial iron detoxification at the molecular level. J Biol Chem 295:17602–17623. doi:10.1074/jbc.REV120.007746 33454001 PMC7762939

[48] Lewis JP, Plata K, Yu F, Rosato A, Anaya C. 2006. Transcriptional organization, regulation and role of the Porphyromonas gingivalis W83 hmu haemin-uptake locus. Microbiology (Reading, Engl) 152:3367–3382. doi:10.1099/mic.0.29011-0 17074906

[49] Lewis JP. 2010. Metal uptake in host–pathogen interactions: role of iron in Porphyromonas gingivalis interactions with host organisms. Periodontol 2000 52:94–116. doi:10.1111/j.1600-0757.2009.00329.x 20017798 PMC2825758

[50] Ireton K, Mortuza R, Gyanwali GC, Gianfelice A, Hussain M. 2021. Role of internalin proteins in the pathogenesis of Listeria monocytogenes*.* Mol Microbiol 116:1407–1419. doi:10.1111/mmi.14836 34704304

[51] Shin J, Choi Y. 2012. The fate of Treponema denticola within human gingival epithelial cells. Mol Oral Microbiol 27:471–482. doi:10.1111/j.2041-1014.2012.00660.x 23134612

[52] Iyer D, Anaya-Bergman C, Jones K, Yanamandra S, Sengupta D, Miyazaki H, Lewis JP. 2010. AdpC is a Prevotella intermedia 17 leucine-rich repeat internalin-like protein. Infect Immun 78:2385–2396. doi:10.1128/IAI.00510-09 20308299 PMC2876575

[53] Sengupta D, Kang DJ, Anaya-Bergman C, Wyant T, Ghosh AK, Miyazaki H, Lewis JP. 2014. Interaction of Prevotella intermedia strain 17 leucine-rich repeat domain protein AdpF with eukaryotic cells promotes bacterial internalization. Infect Immun 82:2637–2648. doi:10.1128/IAI.01361-13 24711565 PMC4019153

[54] Johnston CD, Goetting-Minesky MP, Kennedy K, Godovikova V, Zayed SM, Roberts RJ, Fenno JC. 2023. Enhanced transformation efficiency in Treponema denticola enabled by syngenicDNA-based plasmids lacking restriction-modification target motifs. Mol Oral Microbiol 38:455–470. doi:10.1111/omi.12441 37880921 PMC11024988

[55] Stojkova P, Spidlova P, Stulik J. 2019. Nucleoid-associated protein HU: a lilliputian in gene regulation of bacterial virulence. Front Cell Infect Microbiol 9:159. doi:10.3389/fcimb.2019.00159 31134164 PMC6523023

[56] Priyadarshini R, Cugini C, Arndt A, Chen T, Tjokro NO, Goodman SD, Davey ME. 2013. The nucleoid-associated protein HUβ affects global gene expression in Porphyromonas gingivalis. Microbiology (Reading) 159:219–229. doi:10.1099/mic.0.061002-0 23175503 PMC3709559

[57] Serbanescu MA, Cordova M, Krastel K, Flick R, Beloglazova N, Latos A, Yakunin AF, Senadheera DB, Cvitkovitch DG. 2015. Role of the Streptococcus mutans CRISPR-Cas systems in immunity and cell physiology. J Bacteriol 197:749–761. doi:10.1128/JB.02333-14 25488301 PMC4334182

[58] Henry LG, McKenzie RME, Robles A, Fletcher HM. 2012. Oxidative stress resistance in Porphyromonas gingivalis. Future Microbiol 7:497–512. doi:10.2217/fmb.12.17 22439726 PMC3397238

[59] Mishra A, Aja E, Fletcher HM. 2020. Role of superoxide reductase FA796 in oxidative stress resistance in filifactor alocis. Sci Rep 10:9178. doi:10.1038/s41598-020-65806-3 32513978 PMC7280497

[60] Ohta K, Makinen KK, Loesche WJ. 1986. Purification and characterization of an enzyme produced by Treponema denticola capable of hydrolyzing synthetic trypsin substrates. Infect Immun 53:213–220. doi:10.1128/iai.53.1.213-220.1986 3013780 PMC260099

[61] Fletcher HM, Schenkein HA, Macrina FL. 1994. Cloning and characterization of a new protease gene (prtH) from Porphyromonas gingivalis. Infect Immun 62:4279–4286. doi:10.1128/iai.62.10.4279-4286.1994 7927685 PMC303106

[62] Kurniyati K, Kelly JF, Vinogradov E, Robotham A, Tu Y, Wang J, Liu J, Logan SM, Li C. 2017. A novel glycan modifies the flagellar filament proteins of the oral bacterium Treponema denticola. Mol Microbiol 103:67–85. doi:10.1111/mmi.13544 27696564 PMC5182079

[63] Miyai-Murai Y, Okamoto-Shibayama K, Sato T, Kikuchi Y, Kokubu E, Potempa J, Ishihara K. 2023. Localization and pathogenic role of the cysteine protease dentipain in Treponema denticola. Mol Oral Microbiol 38:212–223. doi:10.1111/omi.12406 36641800 PMC10175099

[64] Patro R, Duggal G, Love MI, Irizarry RA, Kingsford C. 2017. Salmon provides fast and bias-aware quantification of transcript expression. Nat Methods 14:417–419. doi:10.1038/nmeth.4197 28263959 PMC5600148

[65] Sun J, Nishiyama T, Shimizu K, Kadota K. 2013. TCC: an R package for comparing tag count data with robust normalization strategies. BMC Bioinformatics 14:219. doi:10.1186/1471-2105-14-219 23837715 PMC3716788

[66] Klopfenstein DV, Zhang L, Pedersen BS, Ramírez F, Warwick Vesztrocy A, Naldi A, Mungall CJ, Yunes JM, Botvinnik O, Weigel M, Dampier W, Dessimoz C, Flick P, Tang H. 2018. GOATOOLS: a python library for gene ontology analyses. Sci Rep 8:10872. doi:10.1038/s41598-018-28948-z 30022098 PMC6052049

[67] Jumper J, Evans R, Pritzel A, Green T, Figurnov M, Ronneberger O, Tunyasuvunakool K, Bates R, Žídek A, Potapenko A, et al.. 2021. Highly accurate protein structure prediction with AlphaFold. Nature 596:583–589. doi:10.1038/s41586-021-03819-2 34265844 PMC8371605

[68] Mirdita M, Schütze K, Moriwaki Y, Heo L, Ovchinnikov S, Steinegger M. 2022. ColabFold: making protein folding accessible to all. Nat Methods 19:679–682. doi:10.1038/s41592-022-01488-1 35637307 PMC9184281

[69] Varadi M, Bertoni D, Magana P, Paramval U, Pidruchna I, Radhakrishnan M, Tsenkov M, Nair S, Mirdita M, Yeo J, Kovalevskiy O, Tunyasuvunakool K, Laydon A, Žídek A, Tomlinson H, Hariharan D, Abrahamson J, Green T, Jumper J, Birney E, Steinegger M, Hassabis D, Velankar S. 2024. AlphaFold protein structure database in 2024: providing structure coverage for over 214 million protein sequences. Nucleic Acids Res 52:D368–D375. doi:10.1093/nar/gkad1011 37933859 PMC10767828

[70] Ishihara K, Kuramitsu HK. 1995. Cloning and expression of a neutral phosphatase gene from Treponema denticola. Infect Immun 63:1147–1152. doi:10.1128/iai.63.4.1147-1152.1995 7534273 PMC173126

[71] Chen S, Zhou Y, Chen Y, Gu J. 2018. fastp: an ultra-fast all-in-one FASTQ preprocessor. Bioinformatics 34:i884–i890. doi:10.1093/bioinformatics/bty560 30423086 PMC6129281

[72] Barrick JE, Colburn G, Deatherage DE, Traverse CC, Strand MD, Borges JJ, Knoester DB, Reba A, Meyer AG. 2014. Identifying structural variation in haploid microbial genomes from short-read resequencing data using breseq. BMC Genomics 15:1039. doi:10.1186/1471-2164-15-1039 25432719 PMC4300727

[73] Sano Y, Okamoto-Shibayama K, Tanaka K, Ito R, Shintani S, Yakushiji M, Ishihara K. 2014. Dentilisin involvement in coaggregation between Treponema denticola and Tannerella forsythia. Anaerobe 30:45–50. doi:10.1016/j.anaerobe.2014.08.008 25152229

[74] Moffatt-Jauregui CE, Robinson B, de Moya AV, Brockman RD, Roman AV, Cash MN, Culp DJ, Lamont RJ. 2013. Establishment and characterization of a telomerase immortalized human gingival epithelial cell line. J Periodontal Res 48:713–721. doi:10.1111/jre.12059 23441958 PMC3709015

[75] Inagaki S, Onishi S, Kuramitsu HK, Sharma A. 2006. Porphyromonas gingivalis vesicles enhance attachment, and the leucine-rich repeat BspA protein is required for invasion of epithelial cells by “Tannerella forsythia”. Infect Immun 74:5023–5028. doi:10.1128/IAI.00062-06 16926393 PMC1594857

[76] Ishihara K, Kuramitsu HK, Miura T, Okuda K. 1998. Dentilisin activity affects the organization of the outer sheath of Treponema denticola. J Bacteriol 180:3837–3844. doi:10.1128/JB.180.15.3837-3844.1998 9683480 PMC107367

[77] Li H, Kuramitsu HK. 1996. Development of a gene transfer system in Treponema denticola by electroporation. Oral Microbiol Immunol 11:161–165. doi:10.1111/j.1399-302x.1996.tb00352.x 8941770

