# Supplementary material for: Transcription regulator TrtA modulates oxygen stress response in Treponema denticola
Source: Appl Environ Microbiol. 2025 Nov 12;91(12):e00750-25. doi: 10.1128/aem.00750-25 (PMC12724145; doi:10.1128/aem.00750-25)
Supplement: Supplemental material — Tables S1 to S4; Fig. S1. [file aem.00750-25-s0001.docx]

**Supplemental Tables**

**Table S1. Differentially expressed genes in YK7**

| Gene | Annotation | Gene expression  Log_2_(YK7/wild-type) |
| --- | --- | --- |
| TDE_1813 | hypothetical protein | −10.084473 |
| TDE_1788 | conserved hypothetical protein | −8.4971506 |
| TDE_1787 | conserved hypothetical protein | −7.8755309 |
| TDE_0011 | alkyl hydroperoxide reductase/peroxiredoxin | −6.9106524 |
| TDE_2258 | surface antigen BspA, putative | −5.6931619 |
| TDE_2088 | C4 zinc finger domain protein, DksA/TraR family | −5.532164 |
| TDE_2259 | hypothetical protein | −5.1824876 |
| TDE_2059 | hypothetical protein | −4.8995482 |
| TDE_2057 | hypothetical protein | −4.1811428 |
| TDE_2640 | hypothetical protein | −4.1475248 |
| TDE_0742 | hypothetical protein | −4.0259086 |
| TDE_1945 | hypothetical protein | −3.7915207 |
| TDE_2058 | conserved hypothetical protein | −3.7541479 |
| TDE_2055 | hemin-binding protein B | −3.7342196 |
| TDE_2054 | conserved hypothetical protein | −3.6542582 |
| TDE_0850 | methyl-accepting chemotaxis protein | −3.5916688 |
| TDE_2056 | outer membrane hemin-binding protein A | −3.2162629 |
| TDE_2715 | hypothetical protein | −3.1122684 |
| TDE_0946 | hypothetical protein | −3.0612137 |
| TDE_2141 | hypothetical protein | −3.0239411 |
| TDE_1525 | hypothetical protein | −2.9879792 |
| TDE_0012 | carbon starvation protein CstA, putative14.89372567 | −2.9430471 |
| TDE_0969 | conserved hypothetical protein | −2.9298738 |
| TDE_2303 | conserved hypothetical protein | −2.8937738 |
| TDE_0741 | hypothetical protein | −2.8818246 |
| TDE_0328 | CRISPR-associated protein Cas1 | −2.8733418 |
| TDE_2301 | FlhB domain protein | −2.8450008 |
| TDE_1526 | hypothetical protein | −2.8379041 |
| TDE_2366 | high-affinity branched-chain amino acid ABC transporter, permease protein | −2.8325643 |
| TDE_1507 | L-serine dehydratase, iron-sulfur-dependent, beta subunit | −2.8087233 |
| TDE_0327 | CRISPR-associated protein | −2.7688192 |
| TDE_2714 | hypothetical protein | −2.7665671 |
| TDE_1476 | hypothetical protein | −2.7137622 |
| TDE_2543 | hypothetical protein | −2.6732238 |
| TDE_0870 | phosphatase/nucleotidase | −2.6166155 |
| TDE_0697 | hypothetical protein | −2.5994606 |
| TDE_1701 | hypothetical protein | −2.5663381 |
| TDE_2734 | hypothetical protein | −2.5288545 |
| TDE_0949 | enolase | −2.499464 |
| TDE_2365 | high-affinity branched-chain amino acid ABC transporter, permease protein | −2.4814514 |
| TDE_2302 | D domain protein | −2.4756851 |
| TDE_2362 | hypothetical protein | −2.4707684 |
| TDE_0947 | translation elongation factor G, putative | −2.449169 |
| TDE_1506 | L-serine dehydratase, iron-sulfur-dependent, alpha subunit | −2.4417268 |
| TDE_0571 | conserved domain protein | −2.4048398 |
| TDE_2363 | high-affinity branched-chain amino acid ABC transporter, ATP-binding protein | −2.4013358 |
| TDE_0975 | hypothetical protein | −2.3467846 |
| TDE_0948 | conserved domain protein | −2.3460828 |
| TDE_0833 | lipoprotein, putative | −2.3434877 |
| TDE_1383 | hypothetical protein | −2.3393527 |
| TDE_0452 | hypothetical protein | −2.325699 |
| TDE_1527 | hypothetical protein | −2.3210478 |
| TDE_0344 | transcriptional regulator, AbrB family | −2.3006561 |
| TDE_2198 | pyruvate-ferredoxin oxidoreductase | −2.2535955 |
| TDE_0945 | conserved hypothetical protein | −2.2036214 |
| TDE_1029 | Hsp20/alpha crystallin family protein | −2.1952415 |
| TDE_1386 | methyl-accepting chemotaxis protein | −2.1761641 |
| TDE_0973 | DNA repair protein RadC | −2.1727061 |
| TDE_2735 | surface antigen, putative | −2.162867 |
| TDE_2200 | methionine gamma-lyase | −2.1437774 |
| TDE_2199 | racemase, Asp/Glu/Hydantoin family | −2.1195179 |
| TDE_1594 | pyridine nucleotide-disulphide oxidoreductase family protein | −2.1096642 |
| TDE_0418 | lipoprotein, putative | −2.0973065 |
| TDE_1384 | cadmium-translocating P-type ATPase | −2.0910368 |
| TDE_2364 | high-affinity branched-chain amino acid ABC transporter, ATP-binding protein | −2.0900317 |
| TDE_0718 | hypothetical protein | −2.0897565 |
| TDE_0178 | methyl-accepting chemotaxis protein | −2.0822315 |
| TDE_2622 | ABC transporter, ATP-binding protein | −2.0780062 |
| TDE_2021 | hypothetical protein | −2.0576645 |
| TDE_1225 | zinc ABC transporter, ATP−binding protein | −2.0487681 |
| TDE_0153 | coenzyme A disulfide reductase | −2.0421715 |
| TDE_0570 | hypothetical protein | −2.0374712 |
| TDE_0980 | asparaginyl-tRNA synthetase | −1.98611 |
| TDE_1593 | Fe-hydrogenase | −1.9811321 |
| TDE_0832 | hypothetical protein | −1.9794169 |
| TDE_2035 | glucose-1-phosphate adenylyltransferase | −1.9645271 |
| TDE_0423 | hypothetical protein | −1.9635839 |
| TDE_2204 | Na+/H+ antiporter family protein | −1.9347411 |
| TDE_1528 | hypothetical protein | −1.9297204 |
| TDE_0572 | conserved domain protein | −1.9256976 |
| TDE_2522 | ABC transporter, ATP-binding protein | −1.9255319 |
| TDE_0958 | patatin family protein | −1.9239915 |
| TDE_1056 | conserved domain protein | −1.9232286 |
| TDE_2361 | high-affinity branched-chain amino acid ABC transporter, amino acid-binding protein | −1.9207193 |
| TDE_2206 | conserved hypothetical protein | −1.9202635 |
| TDE_0155 | adenylate/guanylate cyclase catalytic domain protein | −1.9117478 |
| TDE_1224 | zinc ABC transporter, permease protein | −1.9055692 |
| TDE_0154 | adenylate/guanylate cyclase catalytic domain protein | −1.8973147 |
| TDE_1324 | hypothetical protein | −1.8680946 |
| TDE_2726 | cyclic nucleotide binding domain/GGDEF domain protein | −1.8498946 |
| TDE_2336 | sodium/dicarboxylate symporter family protein | −1.8453509 |
| TDE_2737 | conserved domain protein | −1.8379306 |
| TDE_2020 | YD repeat protein | −1.8379186 |
| TDE_2360 | dihydroxyacetone kinase family protein | −1.8246379 |
| TDE_2708 | hypothetical protein | −1.820843 |
| TDE_0076 | aldolase, DeoC/FbaB family | −1.7846426 |
| TDE_2119 | glycine reductase complex selenoprotein GrdB2 | −1.7815375 |
| TDE_0956 | conserved hypothetical protein | −1.771734 |
| TDE_2623 | membrane protein, putative | −1.7657348 |
| TDE_0162 | hypothetical protein | −1.7630037 |
| TDE_1072 | lipoprotein, putative | −1.7426168 |
| TDE_1624 | glycine cleavage system P protein, subunit 2 | −1.7298549 |
| TDE_2358 | hypothetical protein | −1.7236028 |
| TDE_1736 | ferredoxin | −1.720589 |
| TDE_0347 | methyl-accepting chemotaxis protein DmcA | −1.7203111 |
| TDE_1702 | hypothetical protein | −1.7184584 |
| TDE_0009 | hypothetical protein | −1.7173097 |
| TDE_0151 | integral membrane protein, YeeE/YedE family | −1.7142373 |
| TDE_2257 | 5′-nucleotidase family protein (protein_id = AAS12776.1) | −1.7079912 |
| TDE_0869 | membrane protein, putative | −1.7058876 |
| TDE_0074 | amino acid permease family protein | −1.7009776 |
| TDE_1223 | zinc ABC transporter, permease protein | −1.6894001 |
| TDE_2256 | membrane protein, putative | −1.6828433 |
| TDE_0078 | glycine reductase complex selenoprotein GrdB1 | −1.680093 |
| TDE_0329 | CRISPR-associated protein Cas2 | −1.6711396 |
| TDE_0955 | LemA family protein | −1.6708096 |
| TDE_0072 | accepting chemotaxis protein | −1.6704744 |
| TDE_1582 | glycogen synthase | −1.6667106 |
| TDE_2626 | ABC transporter, ATP-binding/permease protein | −1.6580318 |
| TDE_0569 | hypothetical protein | −1.6573124 |
| TDE_0080 | betaine aldehyde dehydrogenase | −1.6555414 |
| TDE_0152 | amino acid permease family protein | −1.6555213 |
| TDE_1382 | transcriptional regulator, ArsR family | −1.6523323 |
| TDE_0812 | ABC transporter, ATP-binding/permease protein | −1.6482805 |
| TDE_2359 | DAK2 domain protein | −1.6390584 |
| TDE_1737 | hypothetical protein | −1.6375241 |
| TDE_2050 | hypothetical protein | −1.6335741 |
| TDE_2102 | hypothetical protein | −1.6256134 |
| TDE_1625 | glycine cleavage system P protein, subunit 1 | −1.623343 |
| TDE_2449 | conserved hypothetical protein | −1.6232518 |
| TDE_1703 | hypothetical protein | −1.6168698 |
| TDE_1704 | hypothetical protein | −1.6148782 |
| TDE_1247 | hypothetical protein | −1.6145842 |
| TDE_2493 | conserved domain protein | −1.6123141 |
| TDE_2072 | conserved hypothetical protein | −1.6071718 |
| TDE_2705 | ISTde3, transposase | −1.6059729 |
| TDE_2277 | transcriptional regulator, Sir2 family | −1.5963619 |
| TDE_0957 | glycerophosphoryl diester phosphodiesterase family protein | −1.5879807 |
| TDE_2003 | internalin-related protein | −1.5867816 |
| TDE_2405 | conserved hypothetical protein | −1.5834902 |
| TDE_0860 | ribosomal small subunit pseudouridine synthase A | −1.582932 |
| TDE_2240 | hypothetical protein | −1.5632697 |
| TDE_1705 | hypothetical protein | −1.5630359 |
| TDE_1000 | 3-hydroxyacid dehydrogenase family protein | −1.5600823 |
| TDE_0019 | formate–tetrahydrofolate ligase | −1.5506837 |
| TDE_0398 | oligopeptide/dipeptide ABC transporter, periplasmic peptide-binding protein | −1.5430407 |
| TDE_0077 | glycine reductase complex proprotein GrdE1 | −1.5416714 |
| TDE_0462 | metallo-beta-lactamase family protein | −1.5343457 |
| TDE_2733 | hypothetical protein | −1.5262962 |
| TDE_1628 | hypothetical protein | −1.5109358 |
| TDE_0874 | transcriptional regulator, putative | −1.5091949 |
| TDE_1844 | DNA integrase | −1.502704 |
| TDE_0761 | protease complex-associated polypeptide 13.7117672 | −1.5024011 |
| TDE_1596 | membrane protein, putative | 1.50678783 |
| TDE_1495 | hypothetical protein | 1.51406303 |
| TDE_0487 | ABC transporter, ATP-binding protein | 1.52143463 |
| TDE_1221 | glycosyl hydrolase, family 3 | 1.5270772 |
| TDE_0132 | conserved domain protein | 1.52954836 |
| TDE_1490 | hypothetical protein | 1.53392831 |
| TDE_0341 | ribonucleoside-diphosphate reductase, alpha subunit | 1.54394185 |
| TDE_1271 | oligopeptide/dipeptide ABC transporter, ATP-binding protein | 1.5496659 |
| TDE_1272 | oligopeptide/dipeptide ABC transporter, ATP-binding protein | 1.59346398 |
| TDE_0364 | ABC transporter, ATP-binding protein | 1.59482682 |
| TDE_1915 | alcohol dehydrogenase, iron-containing | 1.61211112 |
| TDE_0546 | hypothetical protein | 1.63304944 |
| TDE_0365 | conserved hypothetical protein | 1.6394979 |
| TDE_1336 | conserved hypothetical protein | 1.68836669 |
| TDE_0750 | magnesium chelatase, subunit D/I family | 1.6999544 |
| TDE_2471 | membrane protein, putative | 1.71532332 |
| TDE_0706 | adenine-specific DNA modification methyltransferase | 1.77761702 |
| TDE_2226 | ABC transporter, substrate-binding protein, putative | 1.778078 |
| TDE_1489 | hypothetical protein | 1.85618902 |
| TDE_0849 | membrane protein, putative | 1.85766868 |
| TDE_2186 | AMP−binding enzyme family protein | 1.86562205 |
| TDE_2620 | conserved hypothetical protein | 1.87796847 |
| TDE_2227 | membrane protein, putative | 1.93344941 |
| TDE_1322 | hypothetical protein | 1.98139307 |
| TDE_0749 | cobalamin biosynthesis protein CobN, putative | 1.98311894 |
| TDE_0414 | hypothetical protein | 1.98802255 |
| TDE_0299 | mutator mutT protein | 2.06601795 |
| TDE_0338 | methyl-accepting chemotaxis protein–like protein 6.137667631 | 2.13165779 |
| TDE_0486 | membrane protein, putative | 2.16182812 |
| TDE_2525 | conserved hypothetical protein | 2.22145493 |
| TDE_0484 | methyl-accepting chemotaxis protein | 2.2774368 |
| TDE_0413 | lipoprotein, putative | 2.32482782 |
| TDE_0755 | Na+/H+ antiporter family protein | 2.3649512 |
| TDE_2262 | peptidase, U32 family | 2.49842422 |
| TDE_1585 | conserved hypothetical protein | 2.50532072 |
| TDE_0485 | membrane protein, putative | 2.51049765 |
| TDE_1586 | conserved hypothetical protein | 2.75438802 |
| TDE_2231 | internalin-related protein | 2.92664017 |

Wild type: *T. denticola* ATCC 35405 phage (-), YK7: *trtA*-deletion mutant of *T. denticola*

Table S2 Mutations in *T. denticola* phage (-) and YK7

| position | mutation | **YK7** | **Wild type** | annotation | gene | description |
| --- | --- | --- | --- | --- | --- | --- |
| 10,168 | A→C |  | 100% | E52A (GAG→GCG) | TDE_0007 → | conserved hypothetical protein |
| 14,124 | T→G | 100% | 100% | H171P (CAT→CCT) | TDE_0009 ← | hypothetical protein |
| 16,534 | +G |  | 100% | coding (227/1455 nt) | TDE_0012 → | carbon starvation protein CstA, putative |
| 21,380 | T→C | 100% |  | Y262H (TAT→CAT) | TDE_0016 → | ATPase, AAA family |
| 30,580 | G→A | 100% |  | S201S (TCC→TCT) | TDE_0024 ← | glycosyl hydrolase, family 3 |
| 30,854 | (T)8→9 | 100% | 100% | coding (329/1674 nt) | TDE_0024 ← | glycosyl hydrolase, family 3 |
| 35,746 | T→C | 100% | 100% | H92R (CAC→CGC) | TDE_0028 ← | ABC transporter, ATP‑binding protein, HlyB family |
| 38,273 | (A)7→6 | 100% | 100% | coding (525/780 nt) | TDE_0030 ← | prolipoprotein diacylglyceryl transferase, putative |
| 46,409 | G→A | 100% |  | P370P (CCC→CCT) | TDE_0040 ← | AMP‑binding protein |
| 50,123 | (T)8→9 | 100% | 100% | coding (296/1884 nt) | TDE_0043 → | TPR domain protein |
| 72,090 | A→G | 100% |  | S218P (TCT→CCT) | pfk ← | phosphofructokinase |
| 84,526 | G→A | 100% |  | T355I (ACA→ATA) | TDE_0074 ← | amino acid permease family protein |
| 85,046 | G→A | 100% |  | Q182* (CAA→TAA) | TDE_0074 ← | amino acid permease family protein |
| 85,474 | C→A | 100% | 100% | G39V (GGG→GTG) | TDE_0074 ← | amino acid permease family protein |
| 86,539 | G→A | 100% |  | T42I (ACA→ATA) | TDE_0075 ← | sorbitol dehydrogenase, putative |
| 86,960 | G→A | 100% |  | L173F (CTT→TTT) | TDE_0076 ← | aldolase, DeoC/FbaB family |
| 95,196 | G→A | 100% |  | A193A (GCC→GCT) | engA ← | GTP‑binding protein EngA |
| 95,310 | G→A | 100% |  | G155G (GGC→GGT) | engA ← | GTP‑binding protein EngA |
| 95,649 | G→A | 100% |  | G42G (GGC→GGT) | engA ← | GTP‑binding protein EngA |
| 103,686 | G→A |  | 100% | P94L (CCT→CTT) | TDE_0086 ← | conserved hypothetical protein |
| 120,198 | G→A | 100% |  | H352Y (CAT→TAT) | TDE_0101 ← | alpha‑amylase family protein |
| 147,828 | (A)8→7 |  | 100% | coding (1449/2406 nt) | TDE_0128 → | HAMP domain/GGDEF domain/EAL domain protein |
| 160,277 | G→A | 100% |  | F799F (TTC→TTT) | TDE_0139 ← | hypothetical protein |
| 171,140 | C→T | 100% | 100% | P188L (CCT→CTT) | TDE_0150 → | ribonuclease BN‑like family protein |
| 180,247 | A→G | 100% |  | intergenic (‑3/+187) | TDE_0155 ← / ← TDE_0156 | adenylate/guanylate cyclase catalytic domain protein/adenylate/guanylate cyclase catalytic domain protein |
| 182,316 | G→T | 100% | 100% | P449H (CCT→CAT) | TDE_0156 ← | adenylate/guanylate cyclase catalytic domain protein |
| 182,320 | A→C | 100% | 100% | C448G (TGT→GGT) | TDE_0156 ← | adenylate/guanylate cyclase catalytic domain protein |
| 182,763 | A→G | 100% | 100% | L300P (CTG→CCG) | TDE_0156 ← | adenylate/guanylate cyclase catalytic domain protein |
| 189,140 | (A)9→8 | 100% | 100% | coding (1103/4905 nt) | TDE_0162 → | hypothetical protein |
| 197,232 | T→C | 100% | 100% | T551A (ACA→GCA) | TDE_0169 ← | methyl‑accepting chemotaxis domain protein |
| 216,429 | (T)6→7 | 100% | 100% | coding (445/1650 nt) | TDE_0182 ← | ABC transporter, ATP‑binding protein |
| 217,747 | T→C | 100% |  | Q312R (CAG→CGG) | TDE_0183 ← | ABC transporter, permease protein |
| 223,993 | (T)6→7 | 100% | 100% | coding (9/768 nt) | TDE_0187 → | carboxylesterase, putative |
| 226,161 | C→T | 100% |  | K130K (AAG→AAA) | TDE_0189 ← | membrane protein, putative |
| 227,264 | (T)8→7 | 100% |  | coding (193/888 nt) | TDE_0190 ← | membrane protein, putative |
| 233,616 | T→C | 100% |  | Y108H (TAT→CAT) | TDE_0198 → | conserved hypothetical protein |
| 240,362 | T→C |  | 100% | E419G (GAG→GGG) | TDE_0206 ← | surface antigen, putative |
| 241,929 | C→T | 100% |  | T63I (ACA→ATA) | TDE_0207 → | permease, GntP family |
| 242,619 | (A)7→8 | 100% |  | coding (878/1356 nt) | TDE_0207 → | permease, GntP family |
| 246,710 | C→T | 100% |  | A223T (GCA→ACA) | TDE_0211 ← | ABC transporter, permease protein, cysTW family |
| 247,329 | (A)8→9 | 100% | 100% | coding (48/732 nt) | TDE_0211 ← | ABC transporter, permease protein, cysTW family |
| 275,034 | C→T | 100% |  | L120L (CTG→CTA) | TDE_0241 ← | membrane protein, putative |
| 277,408 | C→T | 100% |  | G62S (GGC→AGC) | TDE_0243 ← | ABC transporter, ATP‑binding protein |
| 283,918 | C→T | 100% |  | A270T (GCC→ACC) | TDE_0250 ← | sodium‑dependent transporter, putative |
| 283,942 | C→T | 100% |  | D262N (GAT→AAT) | TDE_0250 ← | sodium‑dependent transporter, putative |
| 285,673 | C→T | 100% |  | G172R (GGA→AGA) | tnaA ← | tryptophanase |
| 289,875 | C→T | 100% |  | E320K (GAG→AAG) | TDE_0255 ← | ABC transporter, ATP‑binding protein |
| 290,289 | C→T | 100% |  | V182I (GTT→ATT) | TDE_0255 ← | ABC transporter, ATP‑binding protein |
| 291,870 | C→T | 100% |  | G107R (GGG→AGG) | TDE_0257 ← | membrane protein, putative |
| 306,765 | C→T | 100% |  | intergenic (‑252/‑8) | TDE_0274 ← / → TDE_0275 | ABC transporter, ATP‑binding/permease protein/CAAX amino terminal protease family protein |
| 309,734 | C→A |  | 100% | G47C (GGC→TGC) | TDE_0279 ← | conserved domain protein |
| 317,410 | (A)7→8 | 100% |  | coding (59/1611 nt) | TDE_0286 ← | ABC transporter, ATP‑binding protein |
| 321,362 | C→T | 100% |  | G248D (GGC→GAC) | TDE_0292 ← | conserved hypothetical protein |
| 332,595 | G→C | 100% |  | A37P (GCA→CCA) | TDE_0301 → | tetracenomycin polyketide synthesis O‑methyltransferase TcmP, putative |
| 337,754 | A→G | 100% |  | M319T (ATG→ACG) | sppA ← | signal peptide peptidase SppA |
| 344,507 | A→G | 100% |  | T245A (ACA→GCA) | TDE_0312 → | potassium uptake protein, TrkH family |
| 355,446 | A→G |  | 100% | V17A (GTC→GCC) | TDE_0322 ← | regulatory protein, P‑II family |
| 369,102 | Δ132 bp | 100% |  | intergenic (+2044/+1699) | TDE_0330 → / ← TDE_0331 | CRISPR‑associated protein, SAG0897 family/hypothetical protein |
| 385,619 | T→C | 100% |  | G20G (GGA→GGG) | TDE_0344 ← | transcriptional regulator, AbrB family |
| 406,101 | G→A | 100% | 100% | intergenic (‑167/‑28) | TDE_0360 ← / → TDE_0361 | ABC transporter, ATP‑binding/permease protein/transporter, putative |
| 406,123 | G→T | 100% |  | intergenic (‑189/‑6) | TDE_0360 ← / → TDE_0361 | ABC transporter, ATP‑binding/permease protein/transporter, putative |
| 409,166 | T→C | 100% | 100% | E123E (GAA→GAG) | TDE_0362 ← | bacterial Ig‑like domain protein |
| 419,724 | C→A | 100% |  | E446D (GAG→GAT) | hsdM‑1 ← | type I restriction‑modification system, M subunit |
| 430,025 | A→G | 100% |  | P71P (CCT→CCC) | TDE_0375 ← | ABC transporter, ATP‑binding protein |
| 435,730 | T→C | 100% |  | N181N (AAT→AAC) | TDE_0384 → | hypothetical protein |
| 452,257 | C→A |  | 100% | E688D (GAG→GAT) | TDE_0401 ← | adenylate/guanylate cyclase catalytic domain protein |
| 455,871 | A→G | 100% |  | R88G (AGA→GGA) | TDE_0405 → | major outer sheath protein |
| 457,158 | G→A | 100% | 100% | E517K (GAA→AAA) | TDE_0405 → | major outer sheath protein |
| 457,287 | (T)7→8 | 100% |  | intergenic (+46/‑83) | TDE_0405 → / → TDE_0406 | major outer sheath protein/conserved hypothetical protein |
| 477,443 | (G)5→4 | 100% | 100% | coding (703/1266 nt) | TDE_0423 → | hypothetical protein |
| 479,818 | G→T | 100% | 100% | E603D (GAG→GAT) | TDE_0424 → | bacteriocin‑type signal domain protein |
| 489,006 | T→C | 100% |  | intergenic (‑199/‑43) | TDE_0433 ← / → TDE_0434 | hypothetical protein/rubrerythrin |
| 492,941 | T→C | 100% |  | D252G (GAT→GGT) | TDE_0437 ← | PBS lyase HEAT‑like repeat domain protein |
| 493,174 | C→T |  | 100% | S174S (TCG→TCA) | TDE_0437 ← | PBS lyase HEAT‑like repeat domain protein |
| 497,701 | (T)6→7 | 100% | 100% | coding (113/1035 nt) | TDE_0442 ← | hypothetical protein |
| 500,083 | (A)5→6 | 100% | 100% | coding (200/1281 nt) | TDE_0445 → | amino acid permease |
| 505,190 | (A)7→8 | 100% | 100% | coding (2293/2979 nt) | TDE_0447 → | TPR domain protein |
| 514,742 | T→A | 100% | 100% | L74H (CTT→CAT) | TDE_0459 → | hypothetical protein |
| 515,805 | A→G | 100% |  | G76G (GGT→GGC) | TDE_0460 ← | transporter, putative |
| 516,291 | G→A |  | 100% | P263S (CCT→TCT) | TDE_0462 ← | metallo‑beta‑lactamase family protein |
| 530,513 | (A)7→8 | 100% |  | coding (1753/2100 nt) | TDE_0473 → | heavy metal translocating P‑type ATPase |
| 533,297 | (T)7→8 |  | 100% | coding (1071/1308 nt) | TDE_0477 ← | conserved hypothetical protein |
| 535,428 | G→A |  | 100% | G441G (GGC→GGT) | TDE_0479 ← | hybrid cluster protein |
| 537,491 | C→T | 100% |  | A221A (GCC→GCT) | TDE_0480 → | alpha‑L‑fucosidase family protein |
| 563,387 | A→G | 100% | 100% | W163R (TGG→CGG) | TDE_0515 ← | hypothetical protein |
| 565,042 | T→C | 100% | 100% | T104T (ACA→ACG) | TDE_0518 ← | hypothetical protein |
| 567,135 | G→A | 100% |  | S403L (TCG→TTG) | TDE_0521 ← | carboxylesterase family protein |
| 572,067 | (T)7→6 | 100% | 100% | coding (487/810 nt) | TDE_0526 → | hypothetical protein |
| 577,034 | T→C | 100% |  | C128R (TGC→CGC) | TDE_0534 → | conserved hypothetical protein |
| 586,994 | +G | 100% | 100% | coding (92/114 nt) | TDE_0548 ← | hypothetical protein |
| 589,045 | G→A | 100% | 100% | R147C (CGT→TGT) | TDE_0552 ← | ankyrin repeat protein |
| 595,377 | T→C | 100% |  | S113S (AGT→AGC) | TDE_0562 → | hypothetical protein |
| 599,682 | (T)9→8 | 100% |  | intergenic (+394/‑94) | TDE_0568 → / → TDE_0569 | membrane protein, putative/hypothetical protein |
| 599,722 | (A)10→9 | 100% | 100% | intergenic (+434/‑54) | TDE_0568 → / → TDE_0569 | membrane protein, putative/hypothetical protein |
| 602,575 | C→T | 100% | 100% | T434I (ACA→ATA) | TDE_0571 → | conserved domain protein |
| 607,908 | C→T | 100% |  | G456S (GGT→AGT) | gatA ← | glutamyl‑tRNA(Gln) amidotransferase, A subunit |
| 616,197 | (T)7→8 | 100% | 100% | coding (720/1299 nt) | TDE_0580 → | hypothetical protein |
| 628,203 | (A)7→8 | 100% | 100% | intergenic (+90/+14) | hutH → / ← accA | histidine ammonia‑lyase/acetyl‑CoA carboxylase, carboxyl transferase, alpha subunit |
| 643,091 | T→C | 100% | 100% | H72R (CAC→CGC) | TDE_0605 ← | hypothetical protein |
| 647,549 | (C)13→10 | 100% | 100% | coding (11‑13/105 nt) | TDE_0612 ← | hypothetical protein |
| 650,145 | (G)6→7 | 100% | 100% | intergenic (+6/+38) | TDE_0616 → / ← TDE_0617 | hypothetical protein/hypothetical protein |
| 651,325 | A→G | 100% |  | I290V (ATT→GTT) | TDE_0619 → | methyl‑accepting chemotaxis protein |
| 674,050 | A→T | 100% | 100% | N145K (AAT→AAA) | TDE_0639 ← | oligopeptide/dipeptide ABC transporter, permease protein |
| 682,638 | (A)7→6 | 100% | 100% | coding (555/816 nt) | cheR → | chemotaxis protein methyltransferase |
| 686,722 | +A | 100% | 100% | intergenic (+851/‑1595) | TDE_0650 → / → TDE_0652 | membrane protein, putative/membrane protein, putative |
| 686,805 | T→C | 100% | 100% | intergenic (+934/‑1512) | TDE_0650 → / → TDE_0652 | membrane protein, putative/membrane protein, putative |
| 688,230 | (T)9→10 | 100% |  | intergenic (+2359/‑87) | TDE_0650 → / → TDE_0652 | membrane protein, putative/membrane protein, putative |
| 688,558 | C→A | 100% | 100% | P81H (CCT→CAT) | TDE_0652 → | membrane protein, putative |
| 694,936 | A→G | 100% | 100% | M415V (ATG→GTG) | TDE_0658 → | ABC transporter, permease protein |
| 704,065 | A→G | 100% |  | T125A (ACA→GCA) | sufC → | FeS assembly ATPase SufC |
| 704,305 | G→T | 100% |  | V205F (GTT→TTT) | sufC → | FeS assembly ATPase SufC |
| 713,756 | T→C | 100% |  | T152T (ACT→ACC) | TDE_0674 → | appr‑1‑p processing enzyme family domain protein |
| 736,628 | Δ1 bp | 100% | 100% | coding (710/729 nt) | TDE_0701 ← | membrane protein, putative |
| 738,506 | C→T | 100% | 100% | intergenic (‑226/+13) | TDE_0703 ← / ← TDE_0704 | transcriptional regulator, AbrB family/SPFH domain/Band 7 family protein |
| 740,482 | A→T | 100% | 100% | intergenic (‑124/‑78) | TDE_0705 ← / → TDE_0706 | SPFH domain/Band 7 family protein/adenine‑specific DNA modification methyltransferase |
| 751,128 | A→T | 100% | 100% | E54V (GAA→GTA) | TDE_0716 → | CAAX amino terminal protease family protein |
| 751,558 | C→A | 100% | 100% | A197A (GCC→GCA) | TDE_0716 → | CAAX amino terminal protease family protein |
| 751,907 | 1 bp→TT | 100% | 100% | intergenic (+103/‑12) | TDE_0716 → / → TDE_0717 | CAAX amino terminal protease family protein/hypothetical protein |
| 752,025 | 2 bp→TT | 100% | 100% | coding (107‑108/522 nt) | TDE_0717 → | hypothetical protein |
| 752,038 | T→A | 100% | 100% | L40L (CTT→CTA) | TDE_0717 → | hypothetical protein |
| 752,436 | C→A | 100% | 100% | S173Y (TCC→TAC) | TDE_0717 → | hypothetical protein |
| 763,401 | (C)7→8 | 100% |  | intergenic (+104/‑204) | TDE_0724 → / → TDE_0725 | conserved hypothetical protein/exopolysaccharide biosynthesis protein |
| 778,074 | A→C | 100% | 100% | C59G (TGC→GGC) | TDE_0741 ← | hypothetical protein |
| 778,606 | (GGCTGCGA)9→7 |  | 100% | coding (105‑120/186 nt) | TDE_0742 ← | hypothetical protein |
| 793,916 | A→G | 100% |  | T193A (ACT→GCT) | TDE_0753 → | hypothetical protein |
| 795,873 | (A)6→7 | 100% | 100% | coding (226/1476 nt) | TDE_0755 ← | Na+/H+ antiporter family protein |
| 796,819 | A→G | 100% |  | L44P (CTG→CCG) | TDE_0756 ← | iron compound ABC transporter, ATP‑binding protein, putative |
| 808,668 | G→A | 100% |  | G193S (GGC→AGC) | rplB → | ribosomal protein L2 |
| 838,597 | T→C | 100% |  | T20A (ACT→GCT) | TDE_0816 ← | peptidase, M20/M25/M40 family |
| 852,702 | G→A | 100% | 100% | R5Q (CGG→CAG) | TDE_0832 → | hypothetical protein |
| 853,120 | A→G |  | 100% | G144G (GGA→GGG) | TDE_0832 → | hypothetical protein |
| 856,961 | A→G |  | 100% | L80P (CTT→CCT) | nqrB ← | Na(+)‑translocating NADH‑quinone reductase, B subunit |
| 857,010 | C→T | 100% |  | E64K (GAA→AAA) | nqrB ← | Na(+)‑translocating NADH‑quinone reductase, B subunit |
| 858,484 | T→C | 100% |  | D53G (GAT→GGT) | nqrA ← | Na(+)‑translocating NADH‑quinone reductase, A subunit |
| 869,084 | A→G | 100% |  | S108G (AGC→GGC) | TDE_0846 → | conserved hypothetical protein |
| 875,140 | +T | 100% | 100% | intergenic (‑53/‑19) | TDE_0851 ← / → rplM | hypothetical protein/ribosomal protein L13 |
| 880,586 | (A)8→7 | 100% |  | coding (347/927 nt) | TDE_0859 → | conserved hypothetical protein |
| 880,730 | (A)7→8 | 100% | 100% | coding (491/927 nt) | TDE_0859 → | conserved hypothetical protein |
| 884,937 | C→T |  | 100% | A91V (GCC→GTC) | TDE_0863 → | MgtC family protein |
| 888,765 | (T)8→7 | 100% | 100% | coding (84/1779 nt) | TDE_0867 → | hypothetical protein |
| 896,254 | (A)8→7 | 100% |  | intergenic (+29/‑29) | recA → / → TDE_0873 | recA protein/conserved hypothetical protein |
| 938,839 | (A)7→6 | 100% |  | coding (3423/3627 nt) | TDE_0918 → | hypothetical protein |
| 944,960 | T→C | 100% | 100% | I288V (ATC→GTC) | TDE_0923 ← | ABC transporter, ATP‑binding/permease protein |
| 948,403 | C→T | 100% | 100% | A139A (GCC→GCT) | pepT → | peptidase T |
| 952,826 | A→G | 100% |  | intergenic (‑1/+107) | argF ← / ← TDE_0930 | ornithine carbamoyltransferase/membrane protein, putative |
| 958,773 | G→A | 100% |  | A191T (GCC→ACC) | ackA → | acetate kinase |
| 980,786 | (A)7→8 | 100% | 100% | coding (395/744 nt) | TDE_0957 → | glycerophosphoryl diester phosphodiesterase family protein |
| 980,948 | A→G | 100% |  | Y186C (TAC→TGC) | TDE_0957 → | glycerophosphoryl diester phosphodiesterase family protein |
| 1,006,408 | C→A |  | 100% | P258H (CCC→CAC) | TDE_0981 → | conserved hypothetical protein |
| 1,007,601 | A→G | 100% |  | R329G (AGG→GGG) | TDE_0982 → | dihydroorotate dehydrogenase/oxidoreductase, FAD‑binding |
| 1,007,606 | C→A | 100% | 100% | A330A (GCC→GCA) | TDE_0982 → | dihydroorotate dehydrogenase/oxidoreductase, FAD‑binding |
| 1,008,450 | (T)9→10 | 100% | 100% | intergenic (+58/‑1164) | TDE_0982 → / → TDE_0984 | dihydroorotate dehydrogenase/oxidoreductase, FAD‑binding/oligopeptide/dipeptide ABC transporter, permease protein, putative |
| 1,008,623 | Δ1 bp | 100% | 100% | intergenic (+231/‑991) | TDE_0982 → / → TDE_0984 | dihydroorotate dehydrogenase/oxidoreductase, FAD‑binding/oligopeptide/dipeptide ABC transporter, permease protein, putative |
| 1,013,918 | G→T | 100% | 100% | G240* (GGA→TGA) | TDE_0986 → | oligopeptide/dipeptide ABC transporter, ATP‑binding protein |
| 1,023,715 | C→T | 100% | 100% | P364L (CCC→CTC) | TDE_0996 → | conserved hypothetical protein |
| 1,046,344 | +T | 100% | 100% | intergenic (+685/‑1731) | TDE_1015 → / → xseA | hypothetical protein/exodeoxyribonuclease VII, large subunit |
| 1,047,131 | (A)8→7 | 100% | 100% | intergenic (+1472/‑944) | TDE_1015 → / → xseA | hypothetical protein/exodeoxyribonuclease VII, large subunit |
| 1,051,779 | (T)7→8 | 100% | 100% | coding (905/1029 nt) | TDE_1020 ← | dicarboxylate transporter, periplasmic dicarboxylate‑binding protein, putative |
| 1,084,516 | (T)9→8 | 100% |  | intergenic (‑95/‑834) | TDE_1055 ← / → TDE_1056 | MATE efflux family protein/conserved domain protein |
| 1,088,456 | C→T | 100% | 100% | T557T (ACC→ACT) | TDE_1061 → | ABC transporter, ATP‑binding/permease protein, HlyB family |
| 1,097,010 | C→A | 100% |  | K260N (AAG→AAT) | TDE_1069 ← | oligopeptide/dipeptide ABC transporter, permease protein |
| 1,099,249 | G→T |  | 100% | T477N (ACT→AAT) | oppA ← | peptide ABC transporter, peptide‑binding protein OppA |
| 1,099,408 | A→G |  | 100% | F424S (TTC→TCC) | oppA ← | peptide ABC transporter, peptide‑binding protein OppA |
| 1,100,807 | T→C | 100% | 100% | intergenic (‑129/‑74) | oppA ← / → TDE_1072 | peptide ABC transporter, peptide‑binding protein OppA/lipoprotein, putative |
| 1,101,571 | G→A | 100% | 100% | D231N (GAT→AAT) | TDE_1072 → | lipoprotein, putative |
| 1,101,874 | G→A | 100% |  | A332T (GCC→ACC) | TDE_1072 → | lipoprotein, putative |
| 1,102,034 | C→T | 100% | 100% | S385F (TCT→TTT) | TDE_1072 → | lipoprotein, putative |
| 1,102,196 | C→T | 100% | 100% | A439V (GCC→GTC) | TDE_1072 → | lipoprotein, putative |
| 1,102,231 | 2 bp→GA | 100% | 100% | coding (1351‑1352/2616 nt) | TDE_1072 → | lipoprotein, putative |
| 1,102,553 | A→G | 100% | 100% | Y558C (TAT→TGT) | TDE_1072 → | lipoprotein, putative |
| 1,102,637 | C→T | 100% | 100% | T586I (ACA→ATA) | TDE_1072 → | lipoprotein, putative |
| 1,102,733 | G→T | 100% | 100% | G618V (GGT→GTT) | TDE_1072 → | lipoprotein, putative |
| 1,121,727 | A→G | 100% |  | Y161C (TAC→TGC) | thrS → | threonyl‑tRNA synthetase |
| 1,129,217 | (G)9→12 | 100% | 100% | coding (1013/1578 nt) | TDE_1094 → | conserved hypothetical protein |
| 1,129,695 | G→A | 100% |  | S497S (TCG→TCA) | TDE_1094 → | conserved hypothetical protein |
| 1,138,766 | C→T | 100% | 100% | R736H (CGC→CAC) | infB ← | translation initiation factor IF‑2 |
| 1,146,910 | A→G | 100% |  | I381V (ATT→GTT) | TDE_1111 → | transporter, putative |
| 1,158,064 | A→G | 100% |  | N127S (AAC→AGC) | TDE_1126 → | TPR domain protein |
| 1,164,676 | Δ37,920 bp | 100% | 100% |  | TDE_1133–TDE_1173 | 41 genes |
| 1,203,654 | C→A | 100% |  | G530C (GGT→TGT) | groEL ← | chaperonin, 60 kDa |
| 1,211,623 | (A)8→9 |  | 100% | intergenic (+42/+64) | TDE_1184 → / ← TDE_1185 | hypothetical protein/lipoprotein, putative |
| 1,212,159 | (T)7→8 | 100% | 100% | coding (905/1377 nt) | TDE_1185 ← | lipoprotein, putative |
| 1,216,571 | T→C | 100% | 100% | T48A (ACC→GCC) | pntB ← | NAD(P) transhydrogenase, beta subunit |
| 1,238,961 | A→G | 100% |  | L104L (TTA→TTG) | TDE_1206 → | TPR domain protein |
| 1,258,545 | A→G | 100% |  | G100G (GGT→GGC) | troC ← | zinc ABC transporter, permease protein |
| 1,271,911 | C→T |  | 100% | A131V (GCC→GTC) | TDE_1240 → | PhoH family protein |
| 1,272,674 | T→C | 100% |  | V89A (GTT→GCT) | TDE_1241 → | HD domain protein |
| 1,279,634 | A→G | 100% | 100% | V152V (GTA→GTG) | TDE_1246 → | lipoprotein, putative |
| 1,289,692 | C→T | 100% |  | S19N (AGC→AAC) | TDE_1255 ← | hypothetical protein |
| 1,292,784 | A→G | 100% | 100% | F326S (TTC→TCC) | TDE_1259 ← | amino acid carrier family protein |
| 1,292,860 | C→T | 100% | 100% | A301T (GCC→ACC) | TDE_1259 ← | amino acid carrier family protein |
| 1,292,913 | T→C | 100% | 100% | D283G (GAT→GGT) | TDE_1259 ← | amino acid carrier family protein |
| 1,293,438 | A→G | 100% | 100% | M108T (ATG→ACG) | TDE_1259 ← | amino acid carrier family protein |
| 1,302,498 | (T)5→6 |  | 100% | coding (533/1314 nt) | purD ← | phosphoribosylamine‑‑glycine ligase |
| 1,312,388 | C→T | 100% |  | R164K (AGG→AAG) | TDE_1278 ← | conserved hypothetical protein TIGR01440 |
| 1,332,811 | +C | 100% | 100% | intergenic (‑400/+743) | ptsH ← / ← TDE_1296 | phosphocarrier protein HPr/ribosomal subunit interface protein, putative |
| 1,332,903 | +T | 100% | 100% | intergenic (‑492/+651) | ptsH ← / ← TDE_1296 | phosphocarrier protein HPr/ribosomal subunit interface protein, putative |
| 1,332,918 | +C | 100% | 100% | intergenic (‑507/+636) | ptsH ← / ← TDE_1296 | phosphocarrier protein HPr/ribosomal subunit interface protein, putative |
| 1,340,010 | T→C | 100% |  | intergenic (+2/‑230) | TDE_1304 → / → TDE_1305 | hypothetical protein/DNA‑binding protein |
| 1,358,823 | T→C | 100% |  | L307P (CTA→CCA) | TDE_1320 → | ComEC/Rec2‑related protein |
| 1,363,576 | (A)8→7 | 100% |  | intergenic (‑84/‑67) | TDE_1328 ← / → TDE_1329 | hypothetical protein/ATPase, AAA family |
| 1,372,305 | (A)6→5 | 100% | 100% | coding (1350/1473 nt) | TDE_1335 → | alginate O‑acetylation protein, putative |
| 1,398,230 | (A)7→6 | 100% |  | coding (1025/1368 nt) | TDE_1359 ← | hypothetical protein |
| 1,404,699 | G→A | 100% |  | S9S (AGC→AGT) | TDE_1363 ← | nitroreductase family protein |
| 1,437,833 | A→C | 100% |  | I168L (ATA→CTA) | TDE_1398 → | conserved hypothetical protein |
| 1,446,113 | T→C |  | 100% | T161A (ACG→GCG) | TDE_1403 ← | ABC transporter, ATP‑binding protein |
| 1,451,433 | T→C | 100% | 100% | V155V (GTT→GTC) | TDE_1408 → | flagellar filament outer layer protein FlaA, putative |
| 1,455,461 | (T)7→8 | 100% | 100% | intergenic (+135/‑189) | TDE_1412 → / → TDE_1413 | sodium/hydrogen exchanger family protein/cytidylyltransferase/phosphoenolpyruvate phosphomutase, putative |
| 1,470,204 | (A)9→10 |  | 100% | coding (352/1398 nt) | TDE_1427 → | glycosyl transferase, group 1 family protein |
| 1,472,780 | A→C | 100% | 100% | H66P (CAT→CCT) | TDE_1429 → | glycosyl transferase, group 2 family protein |
| 1,479,630 | Δ1 bp | 100% |  | coding (31/2751 nt) | TDE_1436 → | inner membrane protein |
| 1,502,860 | T→C | 100% | 100% | L428P (CTC→CCC) | TDE_1454 → | sugar transporter |
| 1,524,169 | (A)10→9 |  | 100% | intergenic (‑62/+12) | TDE_1478 ← / ← TDE_1479 | conserved hypothetical protein/DHH superfamily protein, subfamily 1 |
| 1,534,188 | G→A | 100% | 100% | A211V (GCC→GTC) | TDE_1490 ← | hypothetical protein |
| 1,550,808 | T→C | 100% |  | A876A (GCT→GCC) | ppdK → | pyruvate,phosphate dikinase |
| 1,555,840 | (A)9→10 | 100% | 100% | intergenic (‑26/‑276) | sdhB ← / → TDE_1508 | L‑serine dehydratase, iron‑sulfur‑dependent, beta subunit/membrane protein, putative |
| 1,557,153 | C→A | 100% | 100% | P209T (CCC→ACC) | TDE_1509 → | lipoprotein releasing system, ATP‑binding protein, putative |
| 1,557,202 | C→A | 100% | 100% | intergenic (+8/‑118) | TDE_1509 → / → TDE_1510 | lipoprotein releasing system, ATP‑binding protein, putative/membrane protein, putative |
| 1,557,262 | (T)7→8 | 100% |  | intergenic (+68/‑58) | TDE_1509 → / → TDE_1510 | lipoprotein releasing system, ATP‑binding protein, putative/membrane protein, putative |
| 1,570,499 | (C)15→10 | 100% | 100% | coding (68‑72/93 nt) | TDE_1524 → | hypothetical protein |
|  |  |  |  | coding (22‑26/237 nt) | TDE_1525 → | hypothetical protein |
| 1,570,501 | (C)15→12 | Δ | Δ | coding (70‑72/93 nt) | TDE_1524 → | hypothetical protein |
|  |  |  |  | coding (24‑26/237 nt) | TDE_1525 → | hypothetical protein |
| 1,579,372 | T→C | 100% | 100% | Y220C (TAC→TGC) | TDE_1535 ← | conserved hypothetical protein |
| 1,581,343 | (G)6→9 | 100% | 100% | coding (272/1053 nt) | TDE_1537 ← | polyprenyl synthetase family protein |
| 1,593,725 | (A)7→6 | 100% |  | coding (839/1683 nt) | TDE_1550 → | phosphofructokinase, pyrophosphate‑dependent |
| 1,600,853 | (A)8→9 |  | 100% | intergenic (+23/‑44) | TDE_1557 → / → TDE_1558 | hypothetical protein/YD repeat protein |
| 1,604,924 | G→A | 100% |  | W1343* (TGG→TAG) | TDE_1558 → | YD repeat protein |
| 1,645,801 | T→C |  | 100% | E110G (GAG→GGG) | cbiD ← | cobalamin biosynthesis protein CbiD |
| 1,658,830 | A→G | 100% |  | D122G (GAC→GGC) | TDE_1609 → | conserved hypothetical protein |
| 1,676,344 | T→C | 100% | 100% | E53G (GAG→GGG) | gcvT ← | glycine cleavage system T protein |
| 1,701,669 | C→A | 100% | 100% | intergenic (‑1055/‑690) | TDE_1652 ← / → TDE_1654 | ABC transporter, ATP‑binding/permease protein/ATP‑dependent helicase HrpA, putative |
| 1,706,529 | T→C |  | 100% | Q261R (CAG→CGG) | TDE_1656 ← | lipoprotein, putative |
| 1,714,430 | (T)7→8 | 100% | 100% | coding (927/4557 nt) | TDE_1660 ← | leucine Rich Repeat domain protein |
| 1,718,486 | C→T | 100% |  | T162M (ACG→ATG) | TDE_1664 → | conserved domain protein |
| 1,722,975 | (A)7→8 | 100% |  | coding (231/1194 nt) | TDE_1669 → | hemolysin |
| 1,724,848 | T→C | 100% |  | noncoding (67/83 nt) | TDE_tRNA‑Leu‑2 → | tRNA‑Leu |
| 1,731,327 | +C | 100% | 100% | intergenic (‑188/+1686) | atpK ← / ← atpD | V‑type ATPase, K subunit/V‑type ATPase, D subunit |
| 1,735,638 | G→A | 100% |  | P356L (CCC→CTC) | atpA ← | V‑type ATPase, A subunit |
| 1,757,189 | Δ556 bp | 100% | 100% |  | [TDE_1698]–[TDE_1699] | [TDE_1698], [TDE_1699] |
| 1,772,991 | T→C | 100% |  | V231A (GTG→GCG) | guaA → | GMP synthase |
| 1,797,881 | (A)9→8 | 100% |  | intergenic (‑213/+27) | TDE_1742 ← / ← TDE_tRNA‑Pro‑1 | AMP‑binding enzyme family protein/tRNA‑Pro |
| 1,798,867 | A→G | 100% |  | V105A (GTT→GCT) | TDE_1744 ← | chromate transport protein |
| 1,799,819 | T→C | 100% |  | I329M (ATA→ATG) | TDE_1746 ← | conserved hypothetical protein |
| 1,848,379 | G→A | 100% |  | intergenic (‑40/+140) | TDE_1813 ← / ← TDE_1814 | hypothetical protein/hypothetical protein |
| 1,878,520 | G→A | 100% | 100% | Q378* (CAG→TAG) | TDE_1850 ← | ABC transporter, permease protein, putative |
| 1,902,068 | +GC | 100% | 100% | intergenic (‑1233/‑607) | TDE_1878 ← / → TDE_1880 | hydrolase, haloacid dehalogenase‑like family/conserved hypothetical protein |
| 1,918,196 | (T)9→10 |  | 100% | coding (4/585 nt) | purN ← | phosphoribosylglycinamide formyltransferase |
| 1,927,189 | T→C | 100% | 100% | Y580C (TAC→TGC) | dxs ← | 1‑deoxy‑D‑xylulose‑5‑phosphate synthase |
| 1,933,738 | G→A | 100% |  | H374Y (CAT→TAT) | glpK ← | glycerol kinase |
| 1,934,486 | (T)8→9 | 100% | 100% | coding (372/1491 nt) | glpK ← | glycerol kinase |
| 1,947,323 | C→T | 100% |  | E67K (GAA→AAA) | TDE_1929 ← | conserved hypothetical protein |
| 1,962,236 | (A)9→8 | 100% |  | coding (349/1035 nt) | TDE_1946 ← | conserved hypothetical protein |
| 1,963,505 | (TA)3→2 | 100% | 100% | coding (34‑35/933 nt) | TDE_1947 ← | ABC transporter, permease protein |
| 1,965,354 | (T)7→10 | 100% | 100% | coding (804/1524 nt) | TDE_1949 ← | ABC transporter, ATP‑binding protein |
| 1,965,355 | (T)8→7 | 100% | 100% | coding (803/1524 nt) | TDE_1949 ← | ABC transporter, ATP‑binding protein |
| 1,969,159 | T→C | 100% |  | A201A (GCA→GCG) | TDE_1953 ← | transcriptional regulator, TetR family |
| 1,975,979 | G→A | 100% |  | R184K (AGG→AAG) | selB → | selenocysteine‑specific translation elongation factor |
| 1,991,793 | G→A | 100% |  | A19V (GCA→GTA) | TDE_1978 ← | conserved hypothetical protein |
| 2,000,079 | T→C | 100% | 100% | N114S (AAC→AGC) | TDE_1985 ← | hypothetical protein |
| 2,007,114 | G→A | 100% |  | F491F (TTC→TTT) | TDE_1990 ← | ATP‑dependent helicase, DinG family |
| 2,010,448 | (T)6→7 |  | 100% | coding (845/1359 nt) | TDE_1992 → | OmpA family protein |
| 2,019,970 | T→C | 100% |  | A563A (GCA→GCG) | TDE_2001 ← | oligoendopeptidase F, putative |
| 2,021,012 | A→G | 100% |  | L216P (CTT→CCT) | TDE_2001 ← | oligoendopeptidase F, putative |
| 2,036,546 | T→C | 100% | 100% | E359E (GAA→GAG) | TDE_2015 ← | lipoprotein, putative |
| 2,040,796 | G→A | 100% | 100% | S5L (TCG→TTG) | TDE_2019 ← | hypothetical protein |
| 2,047,726 | (T)7→8 | 100% |  | coding (1587/4467 nt) | TDE_2022 ← | YD repeat protein |
| 2,051,220 | +C | 100% | 100% | coding (311/366 nt) | TDE_2025 ← | hypothetical protein |
| 2,058,649 | G→T | 100% | 100% | G86C (GGC→TGC) | TDE_2030 → | lipoprotein, RlpA family |
| 2,086,819 | (A)9→8 | 100% | 100% | coding (159/243 nt) | TDE_2060 → | hypothetical protein |
| 2,090,100 | C→T | 100% |  | E419K (GAG→AAG) | feoB ← | ferrous iron transport protein B |
| 2,093,238 | +C | 100% | 100% | intergenic (+956/‑937) | xseB → / → TDE_2068 | exodeoxyribonuclease VII, small subunit/antigen, putative |
| 2,102,167 | (A)7→8 | 100% |  | coding (2442/3015 nt) | TDE_2075 ← | GGDEF domain/EAL domain protein |
| 2,119,623 | (A)8→7 |  | 100% | coding (771/834 nt) | TDE_2091 → | amino acid ABC transporter, amino acid‑binding protein, putative |
| 2,142,356 | T→G | 100% | 100% | D832A (GAT→GCT) | TDE_2115 ← | HAMP domain protein |
| 2,144,634 | C→T | 100% | 100% | V73I (GTA→ATA) | TDE_2115 ← | HAMP domain protein |
| 2,149,748 | C→A | 100% |  | E536D (GAG→GAT) | TDE_2118 ← | topoisomerase IV, A subunit, putative |
| 2,156,055 | (T)7→6 | 100% |  | intergenic (‑55/‑109) | TDE_2122 ← / → TDE_2123 | DHH superfamily protein/conserved hypothetical protein |
| 2,162,506 | G→A | 100% |  | L90F (CTT→TTT) | TDE_2131 ← | cobalamin biosynthesis protein CbiM, putative |
| 2,163,393 | C→T | 100% |  | G80D (GGC→GAC) | TDE_2132 ← | cobalt ABC transporter, ATP‑binding protein, putative |
| 2,163,813 | (A)8→9 |  | 100% | coding (601/795 nt) | TDE_2133 ← | cobalt transport protein, putative |
| 2,167,976 | C→T | 100% |  | A571V (GCC→GTC) | TDE_2137 → | conserved hypothetical protein |
| 2,170,035 | (A)8→7 | 100% |  | coding (3771/5505 nt) | TDE_2137 → | conserved hypothetical protein |
| 2,177,562 | G→A | 100% |  | V552I (GTT→ATT) | TDE_2142 → | methyl‑accepting chemotaxis protein |
| 2,184,652 | (A)7→8 | 100% |  | intergenic (‑65/+35) | TDE_2147 ← / ← TDE_2148 | lipoprotein, putative/OmpA family protein |
| 2,191,170 | (T)7→8 | 100% | 100% | coding (92/714 nt) | TDE_2157 → | ABC transporter, permease protein, putative |
| 2,193,514 | G→T | 100% | 100% | K62N (AAG→AAT) | TDE_2159 → | membrane protein, putative |
| 2,205,683 | +G |  | 100% | coding (647/1839 nt) | TDE_2176 ← | site‑specific recombinase, putative |
| 2,211,207 | T→C | 100% |  | S298G (AGC→GGC) | trmE ← | tRNA modification GTPase TrmE |
| 2,215,670 | A→G | 100% |  | E111G (GAA→GGA) | TDE_2183 → | conserved hypothetical protein |
| 2,227,885 | T→C | 100% |  | intergenic (‑144/‑16) | pdp ← / → TDE_2192 | pyrimidine‑nucleoside phosphorylase/threonine synthase, putative |
| 2,235,029 | G→A | 100% | 100% | N523N (AAC→AAT) | TDE_2198 ← | pyruvate‑ferredoxin oxidoreductase |
| 2,246,858 | (T)7→8 | 100% | 100% | intergenic (‑134/‑15) | TDE_2207 ← / → TDE_2208 | conserved hypothetical protein/conserved hypothetical protein TIGR00486 |
| 2,255,730 | C→A | 100% | 100% | E338D (GAG→GAT) | mglA ← | galactoside ABC transporter, ATP‑binding protein |
| 2,269,680 | (TA)18→11 | 100% | 100% | intergenic (+167/‑85) | TDE_2230 → / → TDE_2231 | hypothetical protein/internalin‑related protein |
| 2,290,077 | (T)8→9 | 100% |  | coding (498/1263 nt) | uraA → | uracil permease |
| 2,294,511 | G→A | 100% | 100% | G228G (GGC→GGT) | TDE_2257 ← | 5'‑nucleotidase family protein |
| 2,303,272 | (T)7→8 | 100% | 100% | coding (847/987 nt) | TDE_2266 ← | reverse transcriptase family protein |
| 2,306,052 | (A)6→7 | 100% | 100% | intergenic (‑42/+318) | TDE_2269 ← / ← TDE_2270 | conserved hypothetical protein/methyl‑accepting chemotaxis protein |
| 2,306,157 | (G)9→10 | 100% |  | intergenic (‑147/+213) | TDE_2269 ← / ← TDE_2270 | conserved hypothetical protein/methyl‑accepting chemotaxis protein |
| 2,313,856 | (T)9→8 | 100% | 100% | coding (219/264 nt) | TDE_2278 → | hypothetical protein |
| 2,325,266 | +T | 100% | 100% | coding (469/483 nt) | TDE_2288 ← | hypothetical protein |
| 2,325,315 | T→G | 100% | 100% | S140S (TCA→TCC) | TDE_2288 ← | hypothetical protein |
| 2,327,243 | A→G | 100% |  | Q447R (CAG→CGG) | TDE_2289 → | phosphoribulokinase/uridine kinase family protein |
| 2,347,712 | A→G |  | 100% | S160P (TCC→CCC) | TDE_2309 ← | sigma‑54 dependent transcriptional regulator, putative |
| 2,348,026 | G→A |  | 100% | P55L (CCG→CTG) | TDE_2309 ← | sigma‑54 dependent transcriptional regulator, putative |
| 2,361,016 | Δ1 bp | 100% | 100% | coding (925/1146 nt) | TDE_2325 → | conserved hypothetical protein |
| 2,372,064 | C→T | 100% |  | C408Y (TGT→TAT) | TDE_2336 ← | sodium/dicarboxylate symporter family protein |
| 2,372,095 | T→C | 100% | 100% | T398A (ACG→GCG) | TDE_2336 ← | sodium/dicarboxylate symporter family protein |
| 2,373,362 | T→G | 100% | 100% | intergenic (‑76/‑73) | TDE_2336 ← / → TDE_2337 | sodium/dicarboxylate symporter family protein/aminopeptidase |
| 2,397,290 | (T)8→9 | 100% |  | coding (273/510 nt) | TDE_2362 ← | hypothetical protein |
| 2,397,817 | T→C | 100% | 100% | A151A (GCA→GCG) | TDE_2363 ← | high‑affinity branched chain amino acid ABC transporter, ATP‑binding protein |
| 2,444,069 | (T)7→8 | 100% |  | intergenic (‑146/+44) | TDE_2418 ← / ← TDE_2419 | conserved hypothetical protein/conserved hypothetical protein |
| 2,453,261 | C→T | 100% |  | A44T (GCA→ACA) | rplL ← | ribosomal protein L7/L12 |
| 2,454,088 | T→C | 100% | 100% | T13A (ACC→GCC) | rplJ ← | ribosomal protein L10 |
| 2,455,711 | T→C | 100% |  | K60R (AAG→AGG) | nusG ← | transcription antitermination protein NusG |
| 2,461,450 | (T)9→10 |  | 100% | coding (90/2280 nt) | TDE_2431 ← | bacteriocin ABC transporter, ATP‑binding/permease protein, putative |
| 2,469,711 | T→C | 100% |  | A247A (GCA→GCG) | TDE_2439 ← | conserved hypothetical protein |
| 2,472,175 | (AT)3→2 | 100% | 100% | intergenic (‑42/+270) | TDE_2440 ← / ← TDE_2441 | ABC transporter, ATP‑binding/permease protein, putative/hypothetical protein |
| 2,480,803 | T→C | 100% | 100% | W278R (TGG→CGG) | queA → | S‑adenosylmethionine:tRNA ribosyltransferase‑isomerase |
| 2,497,634 | C→A | 100% |  | Q326H (CAG→CAT) | TDE_2475 ← | MATE efflux family protein |
| 2,506,733 | A→G | 100% |  | V6A (GTT→GCT) | TDE_2483 ← | conserved hypothetical protein |
| 2,550,564 | +G | 100% | 100% | coding (514/525 nt) | TDE_2523 → | hypothetical protein |
| 2,555,302 | (A)10→9 | 100% |  | intergenic (‑178/+28) | TDE_2526 ← / ← TDE_tRNA‑Lys‑1 | conserved hypothetical protein/tRNA‑Lys |
| 2,572,916 | Δ1 bp | 100% | 100% | intergenic (‑65/‑101) | TDE_2546 ← / → TDE_2547 | membrane protein, putative/hypothetical protein |
| 2,572,944 | (A)7→8 | 100% | 100% | intergenic (‑93/‑73) | TDE_2546 ← / → TDE_2547 | membrane protein, putative/hypothetical protein |
| 2,572,968 | +A | 100% | 100% | intergenic (‑117/‑49) | TDE_2546 ← / → TDE_2547 | membrane protein, putative/hypothetical protein |
| 2,573,715 | T→C | 100% |  | I97I (ATT→ATC) | TDE_2549 → | methyl‑accepting chemotaxis protein |
| 2,576,922 | G→T | 100% |  | R151S (CGT→AGT) | TDE_2551 ← | hypothetical protein |
| 2,577,710 | C→T | 100% | 100% | G442E (GGG→GAG) | TDE_2552 ← | ABC transporter, ATP‑binding/permease protein |
| 2,584,525 | G→A | 100% | 100% | S318F (TCC→TTC) | TDE_2558 ← | ABC transporter, ATP‑binding/permease protein |
| 2,594,154 | (T)8→7 | 100% | 100% | coding (124/2856 nt) | TDE_2566 ← | conserved domain protein |
| 2,595,147 | (A)8→7 | 100% |  | coding (20/879 nt) | TDE_2567 ← | hypothetical protein |
| 2,602,383 | (A)9→8 | 100% | 100% | coding (572/735 nt) | TDE_2576 → | RNA pseudouridylate synthase family protein |
| 2,611,670 | (A)8→9 | 100% | 100% | coding (749/771 nt) | TDE_2581 → | hypothetical protein |
| 2,621,223 | G→A | 100% |  | G319G (GGC→GGT) | TDE_2586 ← | DNA polymerase III, gamma and tau subunits, putative |
| 2,642,887 | Δ268 bp | 100% | 100% | coding (1272‑1539/2676 nt) | mutS → | DNA mismatch repair protein MutS |
| 2,655,648 | G→T | 100% | 100% | R46M (AGG→ATG) | TDE_2613 → | conserved hypothetical protein |
| 2,657,934 | A→G | 100% |  | W166R (TGG→CGG) | TDE_2616 ← | cyclic nucleotide‑binding protein |
| 2,660,020 | (T)7→8 |  | 100% | coding (489/876 nt) | TDE_2618 → | PHP domain protein |
| 2,668,344 | A→G | 100% |  | V50A (GTA→GCA) | TDE_2626 ← | ABC transporter, ATP‑binding/permease protein |
| 2,674,793 | G→A | 100% |  | R10Q (CGG→CAG) | TDE_2634 → | conserved hypothetical protein |
| 2,676,337 | T→C | 100% |  | L43P (CTG→CCG) | TDE_2636 → | hypothetical protein |
| 2,679,280 | (A)8→9 | 100% | 100% | coding (1021/1890 nt) | pepF → | oligoendopeptidase F |
| 2,679,486 | T→C | 100% | 100% | R409R (CGT→CGC) | pepF → | oligoendopeptidase F |
| 2,686,407 | (T)8→7 |  | 100% | coding (681/996 nt) | TDE_2647 ← | lipoyltransferase and lipoate‑protein ligase family protein |
| 2,691,821 | A→G |  | 100% | T3A (ACA→GCA) | TDE_2654 → | macrolide efflux protein, putative |
| 2,693,989 | G→T |  | 100% | G10C (GGT→TGT) | TDE_2657 → | precorrin‑2 C20‑methyltransferase, putative |
| 2,694,494 | (A)7→6 |  | 100% | coding (533/669 nt) | TDE_2657 → | precorrin‑2 C20‑methyltransferase, putative |
| 2,694,827 | G→A |  | 100% | G65E (GGG→GAG) | TDE_2658 → | cobyrinic acid a,c‑diamide synthase, putative |
| 2,711,036 | C→T |  | 100% | P229P (CCC→CCT) | TDE_2672 → | TPR domain protein |
| 2,718,185 | C→A | 100% | 100% | E432D (GAG→GAT) | TDE_2678 ← | alpha‑amylase family protein |
| 2,719,931 | (A)8→7 | 100% |  | coding (274/885 nt) | TDE_2679 → | conserved hypothetical protein |
| 2,729,545 | (T)9→8 | 100% |  | intergenic (+139/‑16) | TDE_2691 → / → pyrG | hypothetical protein/CTP synthase |
| 2,731,828 | (C)6→9 | 100% |  | coding (556/2802 nt) | TDE_2693 → | ankyrin repeat protein |
| 2,731,829 | (C)7→6 | 100% |  | coding (557/2802 nt) | TDE_2693 → | ankyrin repeat protein |
| 2,731,829 | (C)7→8 |  | 100% | coding (557/2802 nt) | TDE_2693 → | ankyrin repeat protein |
| 2,732,787 | T→C | 100% | 100% | D505D (GAT→GAC) | TDE_2693 → | ankyrin repeat protein |
| 2,752,513 | A→G |  | 100% | H457H (CAT→CAC) | TDE_2706 ← | membrane protein, putative |
| 2,752,551 | T→C | 100% | 100% | I445V (ATT→GTT) | TDE_2706 ← | membrane protein, putative |
| 2,770,598 | A→G |  | 100% | intergenic (‑184/+119) | TDE_2713 ← / ← TDE_2714 | RNA methyltransferase, TrmH family/hypothetical protein |
| 2,779,009 | G→T | 100% |  | A229A (GCC→GCA) | TDE_2721 ← | helicase domain protein |
| 2,813,172 | T→C | 100% | 100% | K146E (AAG→GAG) | TDE_2756 ← | bacterial extracellular solute‑binding protein, family 5 |
| 2,813,436 | A→G | 100% |  | Y58H (TAT→CAT) | TDE_2756 ← | bacterial extracellular solute‑binding protein, family 5 |
| 2,831,685 | (A)7→8 | 100% | 100% | coding (874/1830 nt) | TDE_2781 ← | ABC transporter, ATP‑binding/permease protein |

YK-7: *T. denticola trtA-*deletion mutant, Wild type: *T. denticola* ATCC 35405 phage (-)

**Table S3. Bacterial strains and plasmids used in this study**

| Strain or plasmid | Description | Reference or source | |
| --- | --- | --- | --- |
| Strain |  |  | |
| *Treponema denticola* ATCC 35405 Phage (−) | Wild-type strain, *T. denticola* ATCC 35405, lacks phage region (TDE_1133–TDE_1175) | This study (Supplemental Material 1) | |
| *T. denticola* YK7 | *ermFermAM* insertion mutation in TDE_0127 of ATCC 35405; Em^r^ | This study | |
| *T. denticola* Int-1 | *ermFermAM* insertion mutation in TDE_2231 of ATCC 35405; Em^r^ | This study | |
| *Escherichia coli* TOP10 | Strain used for gene cloning | Takara Bio Inc. | |
| Dam^−^/dcm^−^ *E. coli* | Strain used for amplifying the plasmid for electroporation into *T. denticola* ATCC35405 | New England Biolabs | |
| Plasmid |  |  | |
| pMCL191 | Cm^r^ medium-copy-number plasmid containing MCS and lacZ of pUC19 | (1) | |
| pVA2198 | Em^r^ | (2) | |
| pK127 | Cm^r^ for inactivation of TDE_0127 |  |  |
| pK2231 | Cm^r^ for inactivation of TDE_2231 |  |  |
| pCF693Syn | Cm^r^ for *T. denticola*, Km^r^ for *E. coli* | (3) | |
| pCF0259 | *ermB* promoter and TDE_0259 was inserted into pCF693Syn. Cm^r^ for *T. denticola*, Km^r^ for *E. coli* | (4) | |
| pCF0127 | TDE_0259 was replaced with TDE_0127. Cm^r^ for *T. denticola*, Km^r^ for *E. coli* | This study | |

**Table S4. Primers and Taqman probes used in this study**

| Primer |  |
| --- | --- |
| tde0127U | 5′-CGGCCAGTGAATTCGAAACACGCGGCGCCTTTG-3′ |
| tde0127D | 5′-TCGACTCTAGAGGATTATAGGACGATAAAAATCCGA-3′ |
| pMCL191FW | 5′-ATCCTCTAGAGTCGACCTGCAGGCATGCA-3′ |
| pMCL191RV | 5′-TACCGAGCTCGAATTCACTGGCCGTCGTTT-3′ |
| 127BF | 5′-GATGAGTCTTTAGATAAATATCAACAATTT-3′ |
| 127BR | 5′-AATTAATAATACCTCCAAAATTTG-3′ |
| EMF | 5′-GAGGTATTATTAATTGCTCATCGGTATTTGCAACA-3′ |
| EMR | 5′-ATCTAAAGACTCATCCTACATTCCCTTTAGTAACGTGT-3′ |
| 2231F | 5′-GAGCTCGGTACCCGGTGGCATCGGCCCTTACCG-3′ |
| 2231R | 5′-TGCATGCCTGCAGGTTTTCCCGAACCTCTCGG-3′ |
| 2231VF | 5′-AAAGGTTCTCCTTTGCTTGAGTATAGTCAG-3′ |
| 2231VR | 5′-CTAAAAAGCTCTAAGCCATAAATTAAAAGA-3′ |
| ermB2231F | 5′-CTTAGAGCTTTTTAGCTTTCCAAATTTACAAAAGCG-3′ |
| ermB2231R | 5′-CAAAGGAGAACCTTTAAGTTGTCCCTGAAAAATTTC-3′ |
| pCF-F | 5′-AGGAAACAGCTATGACCATGA-3′ |
| ermBPRR_127 | 5′-TTCATAATCATAACTCATGTAATCACTCCTTCTTAATTACAAAT-3′ |
| pCF_127F | 5′-AGTTATGATTATGAATCTATTTTAGCTAAA-3′ |
| pCF_127R | 5′-TCATAGCTGTTTCCTTTAAAAACAATTGGAGGTGTATTTTTTTAA-3′ |
| Primer and Taqman probe |  |
| 0127F | 5′-GCAATCGAAAACAAACAAAAATGGGT-3′ |
| 0127R | 5′-GGAGTGGTATTGAGTGCATCTGTTA-3′ |
| 0127P | 5′-CCGCCGCAACCTTA-3′ |
| 0011F | 5′-GGATTAAGGAAAAACTGGGTGTAGAGA-3′ |
| 0011R | 5′-CGATTTGGTTTGCAATGCTGTCAT-3′ |
| 0011P | 5′-CCTTCCCTGTAATTGC-3′ |
| 2231F | 5′-TGAAAGAACTTGCGTGCCGATATAA-3′ |
| 2231R | 5′-CAGTGCACCATAAAAATTCCAAAGC-3′ |
| 2231P | 5′-ACGTTGCCCATTTACC-3′ |

F and R: Primer for qRT-PCR, P: Taqman probe for qRT-PCR

**Supplemental Figure**


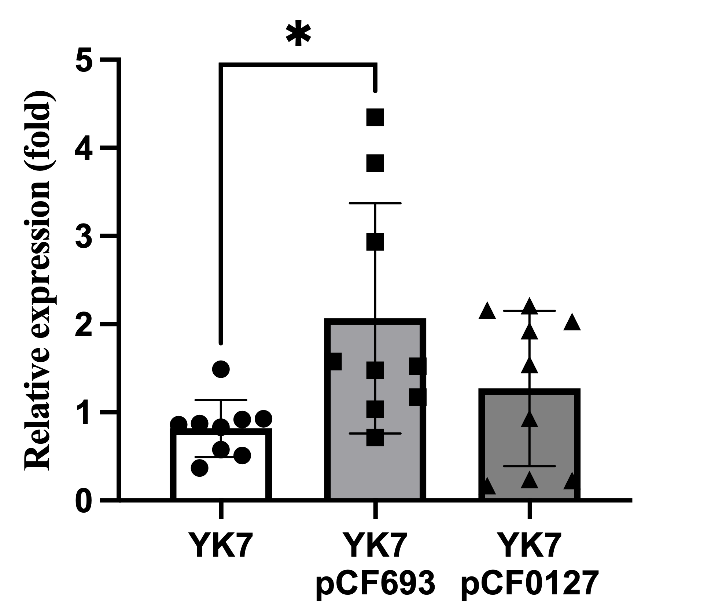


**Fig. S1**

Expression of TDE_0011 in *T. denticola* strains in the mid-log phase was quantified using real-time quantitative reverse transcription PCR.

YK7: *T. denticola trtA*-deletion mutant, YK7/pCF693: *T. denticola* YK7 harboring pCF693Syn (empty vector), YK7/pCF0127: *T. denticola* YK7 harboring pCF0127 (vector carrying *trtA*). The relative expression of TDE_0011 is presented as the mean ± standard deviation (n = 9); statistically significant differences are indicated using asterisks (Student’s t-test; *p < 0.05).

**Supplementary References**

1. Ishihara K, Miura T, Kuramitsu HK, Okuda K. 1996. Characterization of the *Treponema denticola prtP* gene encoding a prolyl-phenylalanine-specific protease (dentilisin). Infect Immun 64:5178–5186.

2. Fletcher HM, Schenkein HA, Macrina FL. 1994. Cloning and characterization of a new protease gene (*prtH*) from *Porphyromonas gingivalis*. Infect Immun 62:4279–4286.

3. Johnston CD, Goetting-Minesky MP, Kennedy K, Godovikova V, Zayed SM, Roberts RJ, Fenno JC. 2023. Enhanced transformation efficiency in *Treponema denticola* enabled by SyngenicDNA-based plasmids lacking restriction-modification target motifs. Mol Oral Microbiol 38(6):455–470.

4. Numata Y, Kikuchi Y, Sato T, Okamoto-Shibayama K, Ando Y, Miyai-Murai Y, Kokubu E, Ishihara K. 2024. Novel transcriptional regulator OxtR1 regulates potential ferrodoxin in response to oxygen stress in *Treponema denticola*. Anaerobe 87:102852.
